# FOXP1 Knockdown Reprograms Th9 CAR‐T Cells to Overcome Antigen Escape

**DOI:** 10.1002/advs.77564

**Published:** 2026-09-16

**Authors:** Yihan Zhu, Xingwei Xie, Xiaohuan Wu, Suidong Ouyang, Yang Zhou, Kang Wen, Yutong Zhong, Yuyang Chen, Handuo Wang, Yuan Gao, Ling Jiang, Hui Li, Wenli Zhao, Abai Xu, Enguang Bi

**Affiliations:** ^1^ Department of Urology Zhujiang Hospital Southern Medical University Guangzhou China; ^2^ Department of Biochemistry and Molecular Biology School of Basic Medical Sciences Southern Medical University Guangzhou Guangdong China; ^3^ Guangdong Provincial Key Laboratory of Medical Immunology and Molecular Diagnostics Guangdong Medical University Dongguan China; ^4^ Department of Hematology Nanfang Hospital Southern Medical University Guangzhou China; ^5^ Hepatology Unit Department of Infectious Diseases Nanfang Hospital Southern Medical University Guangzhou China

**Keywords:** antigen escape, FOXP1, Th9 CAR T cells

## Abstract

Antigen‐loss variants (ALVs) are a major cause of relapse following chimeric antigen receptor (CAR) T cell therapy, particularly in solid tumors where antigen heterogeneity and immune suppression prevail. By integrating public single‐cell RNA sequencing analysis with experimental validation, we identify the transcription factor FOXP1 as a critical brake limiting Th9 CAR‐T cell differentiation and effector programming. FOXP1 knockdown reprograms Th9 CAR‐T but not Tc9 cells toward a metabolically active, cytotoxic, and exhaustion‐resistant phenotype, thereby enhancing their persistence and antitumor activity. CUT&Tag and transcriptomic profiling reveal that FOXP1 binds regulatory regions of *Il9*, *Spi1*, and *Runx1*, as well as effector loci such as *Tnf* and *Gzmb*, repressing both Th9‐lineage and TCR‐downstream transcriptional programs. Its depletion releases this repression, broadly activating MAPK, PI3K‐Akt/mTOR, and NF‐κB pathways that sustain cytokine production and memory formation. Functionally, FOXP1‐deficient Th9 CAR‐T cells eradicate both antigen‐positive and antigen‐loss tumor populations by recruiting dendritic cells and promoting endogenous CD8^+^ T cell clonal expansion via the CD6‐Flt3L axis. Our findings establish FOXP1 as a transcriptional checkpoint integrating cytokine and signaling networks to control Th9 CAR‐T cell function and provide a mechanistic rationale for engineering CAR‐T therapies capable of overcoming antigen escape.

## Graphical abstract

FOXP1 knockdown enhances Th9 CAR‐T cell function by promoting IL‐9 production, effector activation, and reduced exhaustion. Reprogrammed Th9 CAR‐T cells strengthen direct tumor killing and activate endogenous antitumor immunity through dendritic cells and CD8^+^ T cells, thereby suppressing both antigen‐positive and antigen‐loss tumor growth and providing a strategy to overcome antigen escape in CAR‐T therapy.

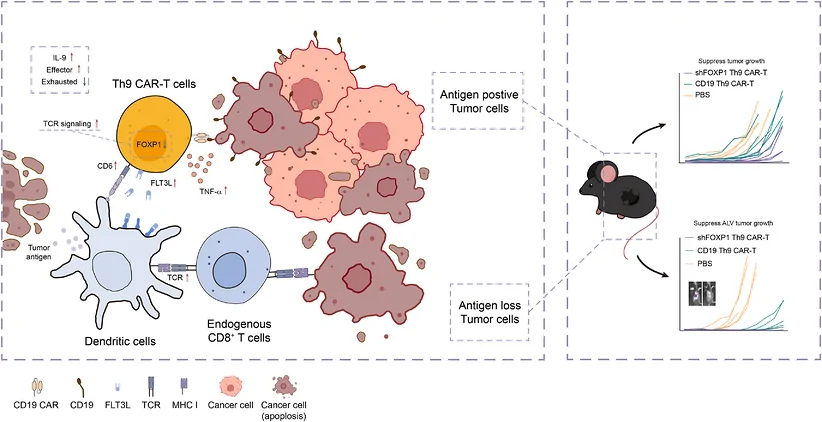

## 1 Introduction

CAR‐T cell therapy, a major breakthrough in adoptive cell transfer (ACT), enables precise recognition and elimination of malignant cells by engineering T cells with synthetic receptors [1, 2]. CAR‐T therapies targeting CD19 and BCMA have revolutionized the treatment of hematological malignancies [3, 4, 5], yet their efficacy against solid tumors remains limited due to multiple immunological and microenvironmental barriers [6, 7]. Among these, tumor antigen loss represents a principal mechanism of immune escape [8, 9]. Under immune selection pressure, tumor cells frequently downregulate or lose target antigens, leading to impaired CAR‐T activity [10], tumor relapse, and therapeutic failure [11, 12, 13]. This challenge is particularly severe in solid tumors, which account for more than 90% of malignancies, where most patients ultimately relapse despite initial responses [11, 14]. To overcome antigen‐loss‐driven resistance, various strategies have been developed, including multi‐antigen targeting, sequential or combinatorial CAR‐T therapy, adapter‐based CAR systems, and bystander‐enhanced CAR‐T designs that secrete immunomodulatory factors [15, 16, 17, 18]. However, approaches capable of eliciting durable bystander immunity remain insufficiently optimized.

CD4^+^ T cells can differentiate into distinct helper subsets under defined cytokine‐polarizing conditions, including Th1, Th2, Th9, Th17, and regulatory T cells (Tregs). Among these subsets, IL‐9‐secreting Th9 cells have demonstrated superior antigen‐specific antitumor activity. Notably, Th9 cells and Th9 CAR‐T cells have recently emerged as promising candidates for overcoming antigen‐loss‐mediated resistance, owing to their capacity to sustain antitumor activity and promote immune responses beyond direct CAR‐mediated recognition [19, 20, 21, 22, 23]. Our previous studies and those of others have demonstrated that Th9‐polarized CAR‐T cells display a central‐memory‐like phenotype, enhanced metabolic fitness, and reduced exhaustion, which together contribute to durable persistence and superior tumor infiltration [21, 24, 25, 26]. Despite these advantages, their therapeutic potential remains constrained by incomplete Th9 differentiation and functional instability. These properties provide a strong rationale for further optimizing Th9 CAR‐T cells to improve tumor control in antigen‐heterogeneous and antigen‐loss settings. We previously identified FOXP1 as a transcriptional repressor of Th9 differentiation, directly suppressing Il9 transcription, whereas FOXO1 counteracts this inhibition by promoting FOXP1 cytoplasmic translocation [25]. Notably, this inhibitory effect of FOXP1 appears to be relatively selective for the Th9 and follicular helper T cell (Tfh) programs, as FOXP1 deficiency has minimal impact on Th1, Th2, or Th17 differentiation [27]. However, it remains unknown whether FOXP1 similarly regulates Th9 CAR‐T differentiation and their capacity to control ALVs, which is a critical question for the design of durable CAR‐T therapies.

In this study, we observed that FOXP1 expression negatively correlates with T cell effector function and clinical response across multiple tumor and CAR‐T cohorts. FOXP1‐high T cells exhibited a quiescent phenotype, whereas FOXP1‐low T cells were enriched for effector and Th9‐related transcriptional programs, supporting its role as a key brake on antitumor immunity. Based on these insights, we engineered FOXP1‐knockdown Th9 CAR‐T cells and evaluated their therapeutic efficacy in antigen positive and antigen‐loss tumor models. FOXP1 silencing enhanced Th9 CAR‐T differentiation, reinforced effector‐like function, and reduced terminal exhaustion both ex vivo and in vivo. Importantly, FOXP1‐deficient Th9 CAR‐T cells mobilized bystander immune activation, promoting endogenous CD8^+^ T cell proliferation and dendritic cell accumulation in tumor‐draining lymph nodes, thereby enabling clearance of antigen‐negative tumor cells and significantly prolonging survival. Collectively, our findings demonstrate that FOXP1 knockdown empowers Th9 CAR‐T cells to overcome antigen‐loss‐driven resistance by strengthening both intrinsic T cell function and host immune engagement, offering a promising strategy to improve ACT efficacy against solid tumors.

## 2 Results

### 2.1 FOXP1 Expression is Negatively Correlated with T Cell Effector Function and Clinical Response

**FIGURE 1:**
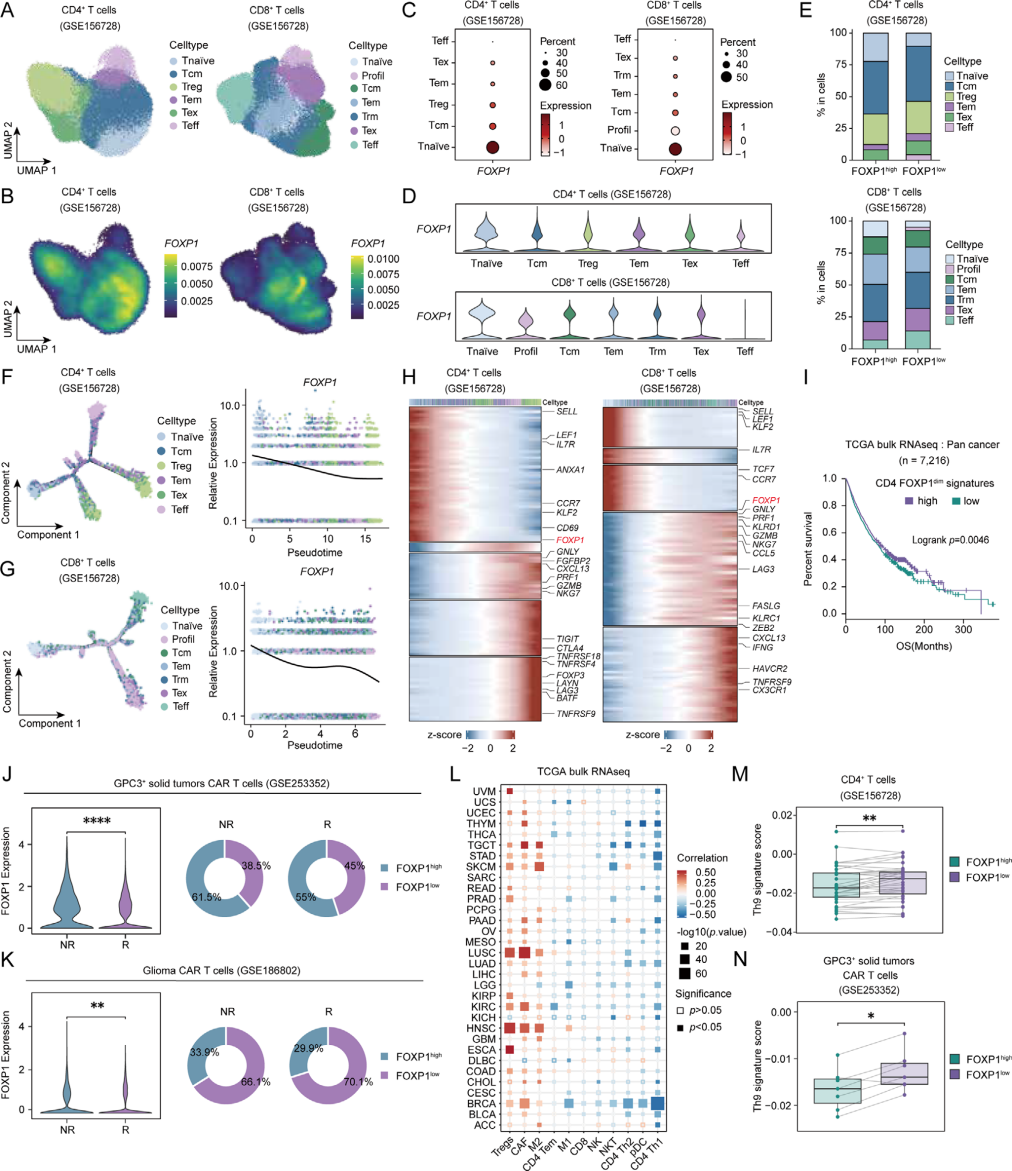
FOXP1 expression is negatively correlated with T cell effector function and clinical response. (A‐H) Single‐cell RNA‐seq analysis from the GSE156728 dataset, including 65,981 CD4^+^ and 95,632 CD8^+^ T cells from seven tumor types: breast cancer (BC), B‐cell lymphoma (BCL), esophageal cancer (ESCA), pancreatic cancer (PACA), renal carcinoma (RC), thyroid carcinoma (THCA), and uterine corpus endometrial carcinoma (UCEC). These are descriptive cell‐level visualizations without pseudobulk aggregation or statistical testing. (A) Uniform Manifold Approximation and Projection (UMAP) plots of CD4^+^ and CD8^+^ T cell subpopulations. (B‐D) Density plot (B), dot plot (C), and violin plot (D) showing *FOXP1* expression across CD4^+^ and CD8^+^ T cell subsets. (E) Comparison of cell‐type composition between FOXP1‐high and FOXP1‐low groups, stratified by normalized *FOXP1* expression (threshold = 2.0). (F‐G) Pseudotime analysis of CD4^+^ (F) and CD8^+^ (G) T cells showing differentiation trajectories (left) and dynamic *FOXP1* expression along pseudotime (right). (H) Heatmap of the top 100 significantly changing genes along pseudotime, ranked by q‐value, with *FOXP1* additionally included. The distribution of cell subpopulations along the pseudotime is shown on top of the heatmap. (I) Kaplan‐Meier survival analysis of patients stratified by enrichment of FOXP1^dim^ CD4^+^ T cell signatures based on bulk RNA‐seq data from the TCGA pan‐cancer cohort (high,n = 3608; low,n = 3,608). (J‐K) *FOXP1* expression levels (left) and proportions of FOXP1‐high and FOXP1‐low CAR‐T cells (right) in responder (R) and non‐responder (NR) patients treated with CD4^+^ CAR‐T therapy, based on GPC3^+^ solid tumor and glioblastoma scRNA‐seq datasets. These analyses were performed at the cell‐level without pseudobulk aggregation. (L) Correlation heatmap between *FOXP1* expression and immune cell‐type abundance across TCGA tumor types derived from TIMER2.0 cell‐type deconvolution, with associations evaluated using purity‐adjusted partial Spearman's correlation; significant correlations (*p* < 0.05) are indicated by solid markers. (M‐N) Patient‐level Th9 signature scores derived from scRNA‐seq data in FOXP1‐high and FOXP1‐low CD4^+^ T cells (M; n = 39 patients) and CD4^+^ CAR T cells (N; n = 7 patients). Within each patient, cells were classified as FOXP1‐high or FOXP1‐low based on the median FOXP1 expression, and the mean Th9 signature score was calculated for each group. Signature scores were summarized at the patient level without pseudobulk aggregation. Each point represents one patient, with paired groups connected by lines. *p < 0.05, **p < 0.01, ***p < 0.001, ****p < 0.0001; two‐sided Wilcoxon rank‐sum test (J, K); two‐sided Wilcoxon signed‐rank test (M, N); a log‐rank test (I).

To define the role of FOXP1 in T cell activation and antitumor immunity, we reanalyzed a large single‐cell RNA sequencing (scRNA‐seq) dataset comprising 161,613 CD3^+^ T cells from seven tumor types with matched T cell receptor (TCR) data [28]. Unsupervised clustering identified major CD4^+^ and CD8^+^ T cell compartments and 13 transcriptionally distinct subsets (Figure 1A). FOXP1 expression was highest in naïve T cells and progressively declined along the differentiation trajectory toward effector states in both CD4^+^ and CD8^+^ lineages (Figure 1B–H). Consistently, FOXP1‐low cells were enriched for effector subsets (Teff), whereas FOXP1‐high cells displayed a quiescent transcriptional profile (Figure 1E). Similar trends were observed in murine tumor‐infiltrating CD4^+^ T cells, where FOXP1 expression decreased as cells transitioned from naïve to Th1‐like effector states (Figure S1A‐G) [29].

To assess the clinical relevance of this transcriptional program at the bulk tumor level, we analyzed bulk RNA‐seq data from the TCGA pan‐cancer cohort. Patients with a high FOXP1^dim^ CD4^+^ T cell signature score exhibited significantly better overall survival (Figure 1I, Table S1). We next examined whether FOXP1 expression was associated with therapeutic response in independent single‐cell RNA‐seq datasets from CAR‐T therapy cohorts targeting GPC3^+^ solid tumors [30] and gliomas [31]. Given the limited number of patients with available response data, these analyses were performed at the individual‐cell level and should therefore be considered exploratory. FOXP1 expression was significantly lower in T cells from responders than in those from non‐responders (Figure 1J,K), supporting a potential association between reduced FOXP1 expression and favorable CAR‐T‐cell therapeutic responses. In a chronic lymphocytic leukemia cohort [32] analyzed by bulk RNA‐seq, a higher FOXP1^dim^ CD4^+^ T cell signature score in CAR‐T cells was associated with better response, and lower FOXP1 expression showed a trend toward prolonged survival (Figure S1H,I, Table S1). Cell‐level analyses across multiple scRNA‐seq immune checkpoint blockade (ICB) cohorts [33, 34, 35, 36], high FOXP1 expression was consistently associated with poor treatment response (Figure S1J). Furthermore, deconvolution analysis of TCGA bulk RNA‐seq data revealed that higher FOXP1 expression correlated with increased infiltration of immunosuppressive populations such as Tregs, M2 macrophages, and cancer‐associated fibroblasts (CAFs), whereas lower FOXP1 expression correlated with greater infiltration of cytotoxic subsets, including CD8^+^ T cells, natural killer (NK) cells, and M1 macrophages (Figure 1L, Table S2) [37].

Together, these data suggest that FOXP1 is associated with the maintenance of a quiescent T cell state, whereas its downregulation promotes effector differentiation and antitumor immunity. Consistent with our previous findings and those of others showing that FOXP1 expression is negatively correlated with Th9 differentiation and Th9‐mediated tumor control [21, 24, 25], we further found in the present study that patient‐level analysis of scRNA‐seq data showed higher IL‐9 signature scores in FOXP1‐low T cells within the tumor microenvironment (Figure 1M,N, Table S1), linking FOXP1 suppression to activation of the Th9 program and providing a rationale for targeting FOXP1 to potentiate Th9 CAR‐T cell function in tumor control.

### 2.2 FOXP1 Knockdown Effectively Drives Th9 CAR‐T Differentiation

**FIGURE 2:**
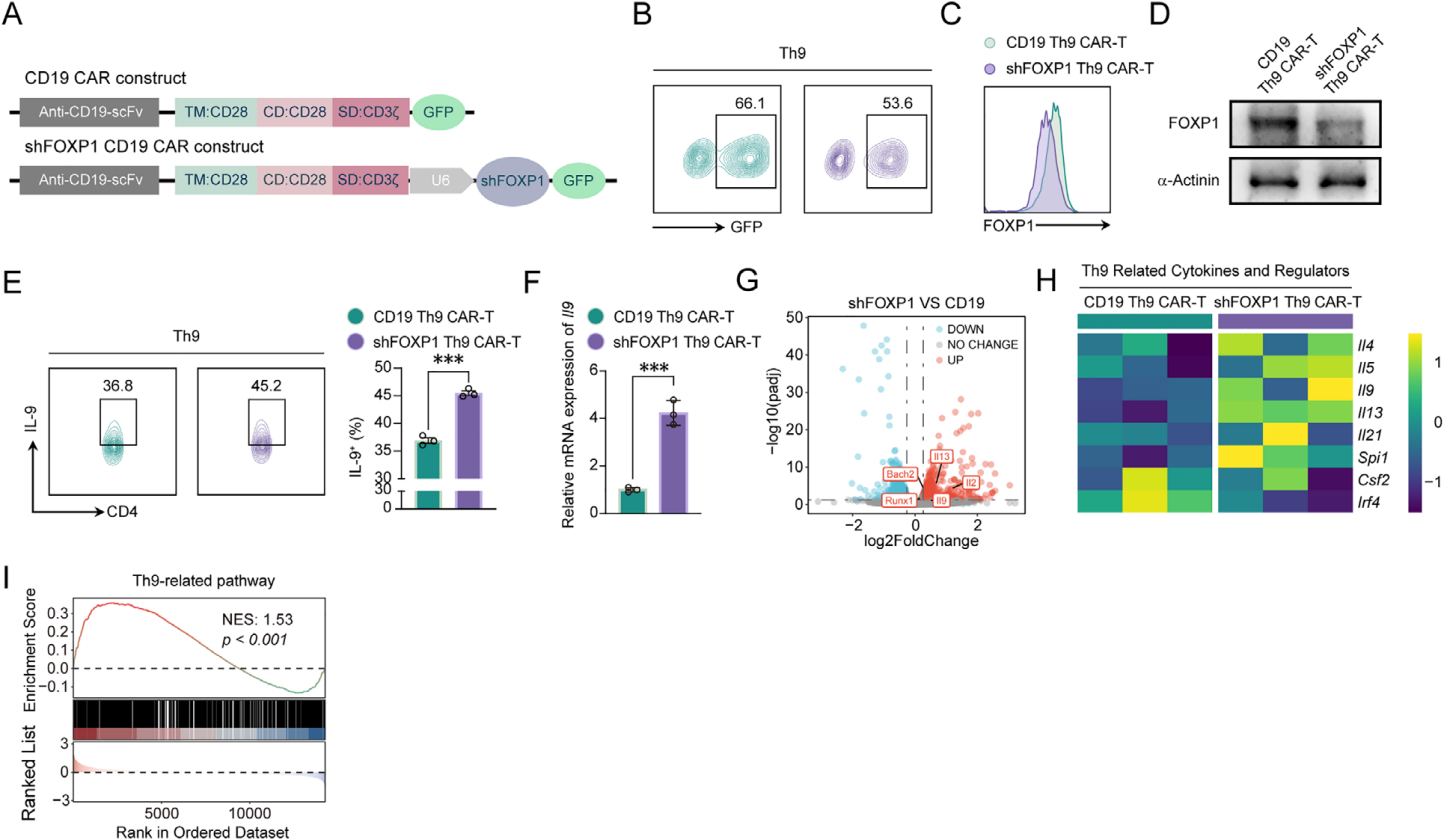
FOXP1 knockdown promotes Th9 differentiation in CAR‐T cells. (A) Schematic representation of the retroviral plasmid constructs used to generate CD19 Th9 CAR‐T and shFOXP1 Th9 CAR‐T cells. (B) Flow cytometric analysis of GFP^+^ transduction efficiency following retroviral construction of shFOXP1 Th9 CAR‐T and CD19 Th9 CAR‐T cells. (C) Flow cytometric validation of FOXP1 knockdown efficiency in shFOXP1 Th9 CAR‐T cells compared with CD19 Th9 CAR‐T cells. (D) FOXP1 knockdown efficiency in shFOXP1 Th9 CAR‐T cells compared with CD19 Th9 CAR‐T controls, determined by Western blot analysis. (E) Flow cytometric measurement of interleukin‐9 (IL‐9) secretion in CD19 Th9 CAR‐T and shFOXP1 Th9 CAR‐T cells, with corresponding quantitative analysis. Mean ± SEM, n = 3. (F) mRNA expression of *Il9* in CD19 Th9 CAR‐T and shFOXP1 Th9 CAR‐T cells. Mean ± SEM, n = 3. (G) Differential gene expression profile between shFOXP1 Th9 CAR‐T and CD19 Th9 CAR‐T cells obtained by bulk RNA‐seq. (H) Expression of Th9‐related cytokines and regulators in CD19 Th9 CAR‐T and shFOXP1 Th9 CAR‐T cells obtained by bulk RNA‐seq. (I) GSEA of bulk RNA‐seq data comparing shFOXP1 Th9 CAR‐T and CD19 Th9 CAR‐T cells, showing enrichment of Th9‐related pathways. NES, normalized enrichment score. *p < 0.05, **p < 0.01, ***p < 0.001; two‐tailed unpaired t test (E, F).

Guided by these observations, we next examined whether FOXP1 downregulation could directly promote Th9 CAR‐T differentiation. FOXP1‐knockdown CAR‐T cells (shFOXP1 Th9 CD19 CAR‐T) were generated using a retroviral vector encoding FOXP1‐targeting shRNA (Figure 2A). Th9 CAR‐T cells were efficiently transduced, achieving over 50% GFP positivity (Figure 2B). In GFP^+^ cells, FOXP1 knockdown was verified by flow cytometry (Figure 2C) and further confirmed by western blot analysis of sorted GFP^+^ CAR‐T cells (Figure 2D).

FOXP1 depletion markedly enhanced Th9 polarization, as shFOXP1 groups secreted significantly higher IL‐9 and expressed more mRNA than controls (Figure 2E,F). RNA‐seq analysis revealed selective upregulation of Th9‐related cytokines and transcriptional regulators and enrichment of the Th9‐specific signaling pathway in shFOXP1 groups (Figure 2G–I) [24].

Collectively, these findings demonstrate that FOXP1 knockdown reprograms CAR‐T cells toward a Th9‐like phenotype, activating a type 2 cytokine transcriptional program that may strengthen their antitumor potential.

### 2.3 FOXP1 Knockdown Enhances the Cytotoxic Activity of Th9 CAR‐T Cells In Vitro

**FIGURE 3:**
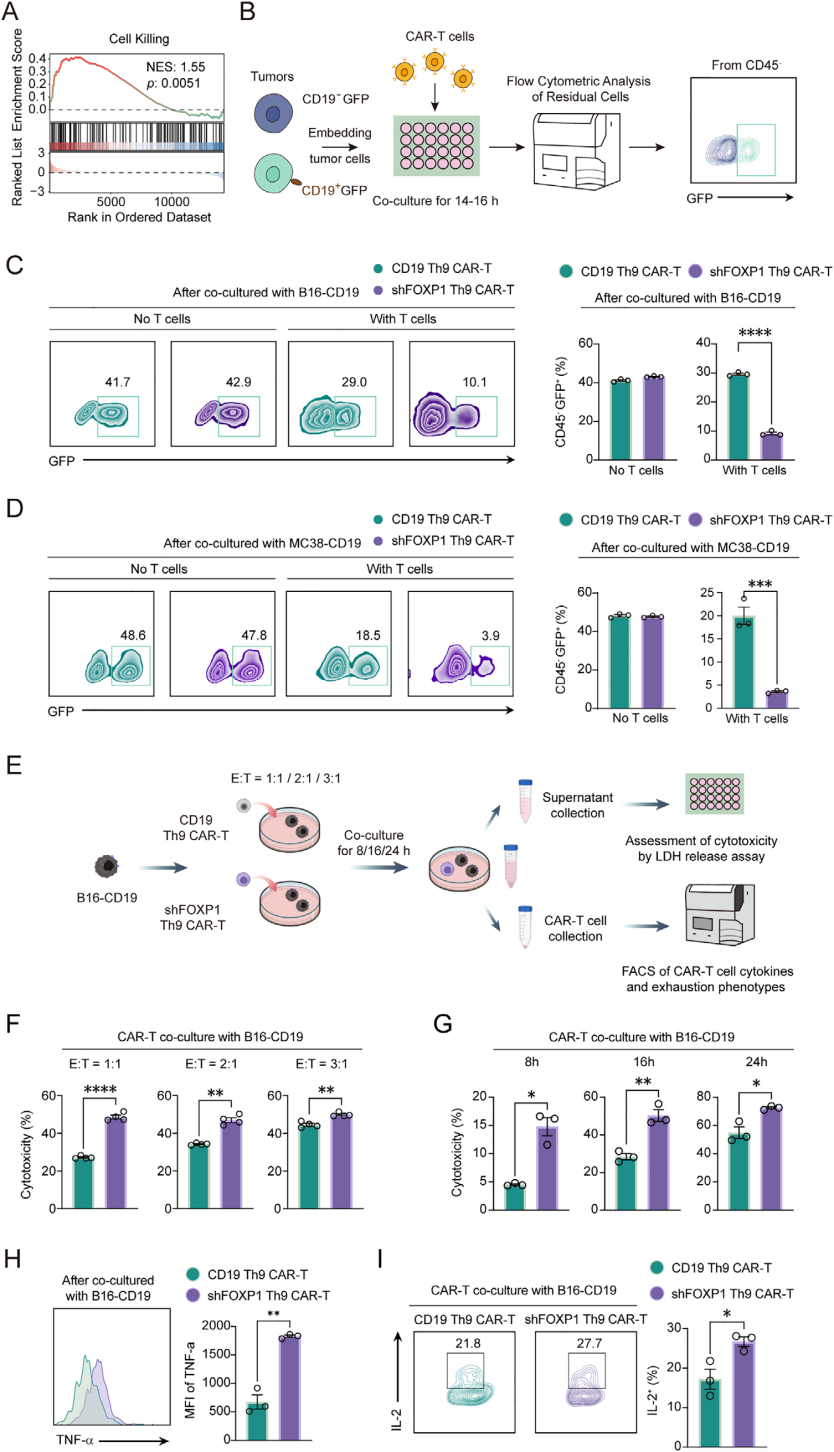
FOXP1 knockdown enhances the in vitro antitumor activity of Th9 CAR‐T cells. (A) GSEA of bulk RNA‐seq data comparing shFOXP1 Th9 CAR‐T and CD19 Th9 CAR‐T cells showing enrichment of cell killing‐related gene sets. (B) Schematic of the flow cytometry‐based tumor‐cell killing assay. CAR‐T cells were co‐cultured with a 1:1 mixture of CD19^−^GFP^−^ parental tumor cells and CD19^+^GFP^+^ target tumor cells, including B16 WT/B16‐CD19 or MC38 WT/MC38‐CD19 cells, for 14–16 h. Residual tumor cells were analyzed by flow cytometry after first gating on CD45^−^ cells to exclude CAR‐T cells, and the percentage of GFP^+^ cells within the CD45^−^ population was used to quantify remaining CD19^+^ target tumor cells. (C) Remaining tumor cell ratios after co‐culture of CD19 Th9 CAR‐T or shFOXP1 Th9 CAR‐T cells with B16‐CD19 tumor cells at E:T ratios of 0:1 (tumor cells only, negative control) and 4:1. Mean ± SEM, n = 3. (D) Cytotoxicity of CD19 Th9 CAR‐T or shFOXP1 Th9 CAR‐T cells against MC38‐CD19 tumor cells at E:T ratios of 0:1 and 1:1. Mean ± SEM, n = 3. (E) Schematic of the LDH release‐based cytotoxicity assay. B16‐CD19 tumor cells were co‐cultured with CD19 Th9 CAR‐T or shFOXP1 Th9 CAR‐T cells at the indicated E:T ratios of 1:1, 2:1, and 3:1 for 8, 16, or 24 h. Culture supernatants were collected for LDH release assay, and CAR‐T cells were collected for flow cytometric analysis of cytokines and exhaustion phenotypes. (F) LDH release‐based cytotoxicity assay comparing CD19 Th9 CAR‐T and shFOXP1 Th9 CAR‐T cells after co‐culture with B16‐CD19 tumor cells at the indicated E:T ratios. Cytotoxicity was assessed after 16 h of co‐culture. Mean ± SEM, n = 4. (G) LDH release‐based cytotoxicity assay comparing CD19 Th9 CAR‐T and shFOXP1 Th9 CAR‐T cells after co‐culture with B16‐CD19 tumor cells for the indicated time points. The E:T ratio was 1:1. Mean ± SEM, n = 3. (H) Flow cytometric analysis of TNF‐α expression in CD19 Th9 CAR‐T and shFOXP1 Th9 CAR‐T cells after co‐culture. Mean ± SEM, n = 3. (I) Flow cytometric analysis of IL‐2 expression in CD19 Th9 CAR‐T and shFOXP1 Th9 CAR‐T cells after co‐culture. Mean ± SEM, n = 3. *p < 0.05, **p < 0.01, ***p < 0.001, ****p < 0.0001; two‐tailed unpaired t test (C, D, F, G, H, I).

Given that Th9 CAR‐T cells exhibit superior antitumor potential compared with other T‐helper subsets, we next investigated whether FOXP1 knockdown‐induced Th9 differentiation enhances their cytotoxic function in vitro. Gene set enrichment analysis (GSEA) revealed significant upregulation of cell killing signaling pathways in shFOXP1 groups (Figure 3A), suggesting that FOXP1 silencing may potentiate killing‐associated programs. Consistently, pseudobulk GSEA and patient‐ or sample‐level signature analyses of independent murine and human scRNA‐seq datasets showed enrichment of immune cytotoxic pathways and higher cytotoxicity signature scores in FOXP1‐low CD4^+^ T cells (Figure S2A–F, Table S1) [38, 39], supporting a conserved link between FOXP1 repression and effector activation.

To directly assess tumor killing, we co‐cultured Th9 CAR‐T cells with GFP‐labeled target tumor cells (Figure 3B). In the B16‐CD19 melanoma model, the residual proportion of CD45^−^ GFP^+^ tumor cells was markedly reduced in the shFOXP1 groups compared with controls (10.1% vs. 29.0%) (Figure 3C). Similar results were obtained in the MC38‐CD19 colon cancer model, confirming that FOXP1 knockdown confers stable and reproducible cytotoxic enhancement (Figure 3D). To further validate this effect using an independent cytotoxicity assay, we performed LDH release assays across multiple effector‐to‐target ratios and co‐culture time points. Consistently, shFOXP1 Th9 CAR‐T cells exhibited increased tumor‐cell killing compared with CD19 Th9 CAR‐T cells under the tested conditions (Figure 3E–G).

To further connect pathway activation with effector function, flow cytometric analysis revealed that shFOXP1 groups expressed significantly higher levels of TNF‐α after co‐culture (Figure 3H), consistent with enrichment of cell killing pathways (Figure 3A). In addition, IL‐2 production was increased in shFOXP1 Th9 CAR‐T cells compared with CD19 Th9 CAR‐T cells (Figure 3I), further supporting enhanced functional activation of FOXP1‐deficient Th9 CAR‐T cells.

Collectively, these findings demonstrate that FOXP1 knockdown strengthens the cytotoxic machinery of Th9 CAR‐T cells, linking FOXP1 repression to activation of effector and cell killing programs that underlie enhanced tumor‐cell clearance in vitro.

### 2.4 FOXP1 Knockdown Promotes Effector‐Memory Differentiation and Alleviates Exhaustion in Th9 CAR‐T Cells

**FIGURE 4:**
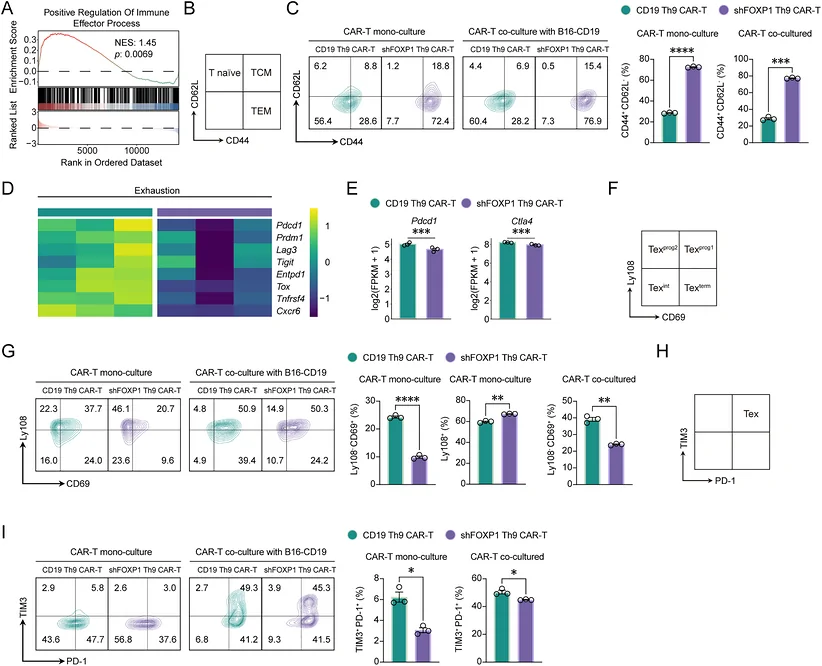
FOXP1 knockdown promotes effector memory differentiation in Th9 cells. (A) GSEA of bulk RNA‐seq comparing shFOXP1 Th9 CAR‐T cells with CD19 Th9 CAR‐T cells, showing enrichment of the immune effector process gene set. (B‐C) Flow cytometric analysis of CD44 and CD62L expression in CD19 Th9 CAR‐T and shFOXP1 Th9 CAR‐T cells under mono‐culture conditions or after co‐culture with B16‐CD19 tumor cells. Mean ± SEM, n = 3. (D) Bulk RNA‐seq analysis of exhaustion‐associated gene expression in CD19 Th9 CAR‐T and shFOXP1 Th9 CAR‐T cells. (E) Bulk RNA‐seq analysis showing normalized expression of *Pdcd1* and *Ctla4* in CD19 Th9 CAR‐T and shFOXP1 Th9 CAR‐T cells. Mean ± SEM, n = 3. (F‐G) Flow cytometric analysis of CD69 and Ly108 expression in CD19 Th9 CAR‐T and shFOXP1 Th9 CAR‐T cells under mono‐culture conditions or after co‐culture with B16‐CD19 tumor cells. Mean ± SEM, n = 3. (H‐I) Flow cytometric quantification of PD‐1 and TIM‐3 expression in CD19 Th9 CAR‐T and shFOXP1 Th9 CAR‐T cells under mono‐culture conditions or after co‐culture with B16‐CD19 tumor cells. Mean ± SEM, n = 3. *p < 0.05; **p < 0.01; ***p < 0.001; ****p < 0.0001; two‐tailed unpaired t test (C, E, G, I).

Because the cytotoxic activity of CAR‐T cells largely depends on effector populations, we next investigated whether FOXP1 knockdown alters the differentiation landscape of Th9 CAR‐T cells. Transcriptomic analysis revealed significant enrichment of pathways associated with the positive regulation of immune effector processes in shFOXP1 groups compared with controls (Figure 4A). Consistently, pseudobulk GSEA and patient‐level signature analyses showed enhanced effector programs in FOXP1‐low CD4^+^ T cells across murine and human cancer scRNA‐seq datasets (Figure S3A–C, Table S1) [38, 39], supporting a conserved role of FOXP1 repression in promoting effector programming.

Flow cytometric analysis of CD44 and CD62L expression demonstrated a shift toward an effector‐memory phenotype (CD62L^−^CD44^+^) in shFOXP1 groups relative to controls both mono‐culture and tumor co‐culture conditions (Figure 4B,C). This phenotypic transition mirrors the transcriptomic data, indicating that FOXP1 knockdown drives T cells from a quiescent toward an activated effector‐memory state, potentially enhancing cytotoxic function.

Because T cell exhaustion critically limits CAR‐T persistence, we next examined exhaustion‐associated profiles. shFOXP1 groups exhibited reduced expression of canonical exhaustion markers, including *Pdcd1*, *Ctla4*, *Lag3*, and *Tigit*, compared with controls (Figure 4D,E). Flow cytometric analysis based on a four‐stage exhaustion model (Ly108/CD69) further revealed a marked decrease in terminally exhausted (Ly108^−^CD69^+^) populations under both mono‐culture and tumor co‐culture conditions (Figure 4F,G). Similarly, the frequency of TIM‐3^+^PD‐1^+^ double‐positive cells was significantly lower in shFOXP1 groups under both conditions (Figure 4H,I), confirming a less exhausted phenotype.

Collectively, these results demonstrate that FOXP1 knockdown intrinsically reprograms Th9 CAR‐T cells toward an effector‐memory phenotype with reduced exhaustion.

### 2.5 FOXP1 Knockdown Enhances the Persistence and Antitumor Efficacy of Th9 CAR‐T Cells In Vivo

**FIGURE 5:**
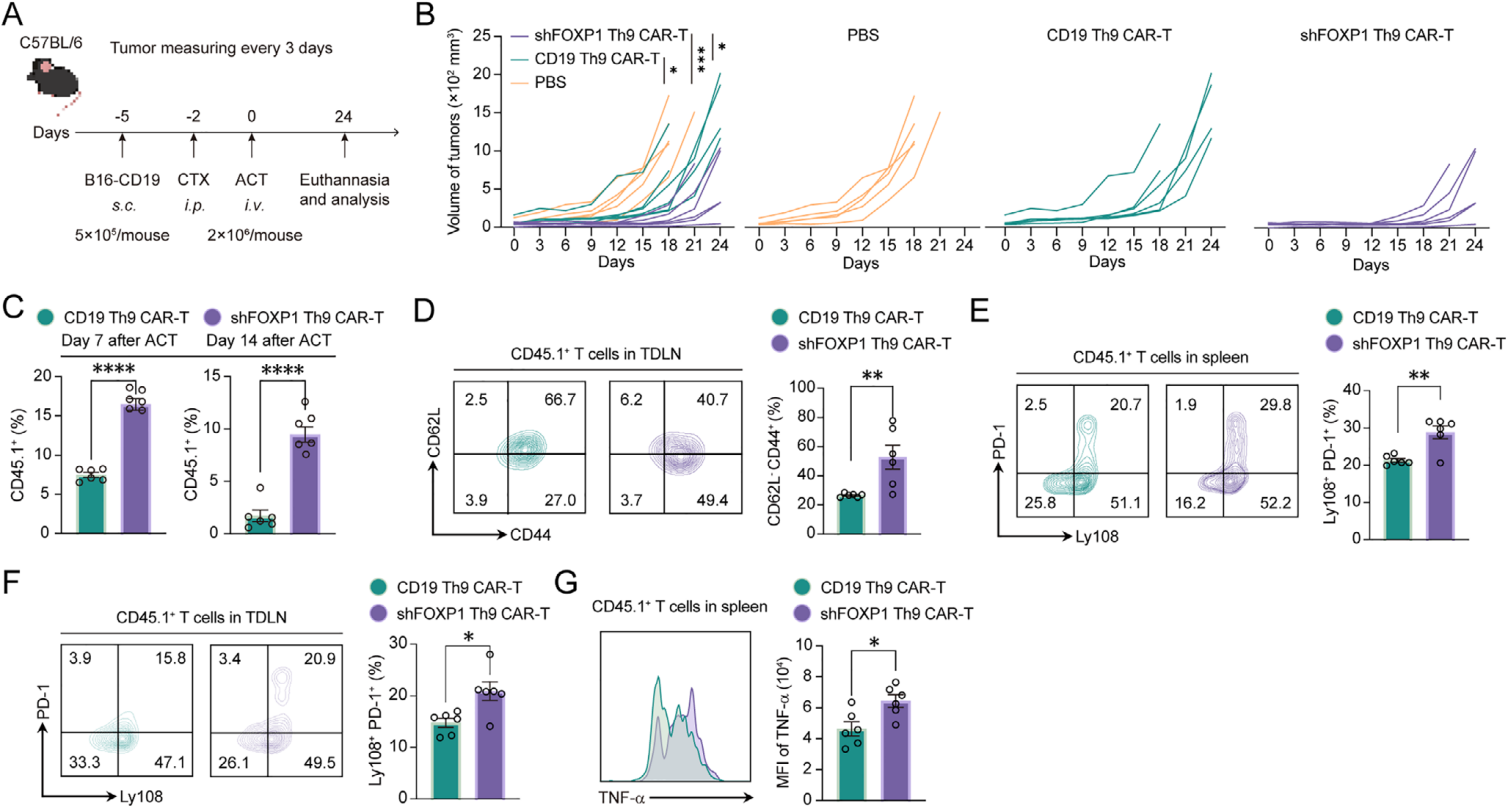
shFOXP1 Th9 CAR‐T cells demonstrate superior in vivo antitumor efficacy. (A) Schematic of the in vivo experimental design. C57BL/6 mice were subcutaneously injected with 5×10^5^ B16‐CD19 tumor cells. Three days after tumor inoculation, cyclophosphamide (CTX) was administered intraperitoneally to deplete endogenous immune cells. Within 48 h of CTX treatment, mice were randomized into three groups and infused intravenously with 2×10^6^ Th9 CAR‐T cells (group PBS, n = 5; group CD19 Th9 CAR‐T, n = 6; group shFOXP1 Th9 CAR‐T, n = 6). (B) Tumor burden was measured with vernier calipers every 3 days after Th9 CAR‐T cell infusion. Mean ± SEM. (C) Frequencies of CD45.1^+^ cells in peripheral blood were analyzed by flow cytometry on days 7 and 14 post‐CAR‐T cell infusion. Mean ± SEM, n = 6. (D) Representative flow cytometry plots showing the percentage of CD44^+^CD62L^−^ effector memory (TEM) cells among infused Th9 CAR‐T cells in tumor‐draining lymph nodes on day 24 post‐treatment. Mean ± SEM, n = 6. (E‐F) Representative flow cytometry plots from spleen (E) and tumor‐draining lymph nodes (F) showing the percentage of Ly108^+^PD‐1^+^ cells among infused Th9 CAR‐T cells on day 24 post‐treatment. Mean ± SEM, n = 6. (G) Representative flow cytometry plots showing TNF‐α expression in CD45.1^+^ CAR‐T cells isolated from spleen on day 24 post‐transfer. Mean ± SEM, n = 6. *p < 0.05; **p < 0.01; ***p < 0.001; ****p < 0.0001, two‐tailed unpaired t test (B, C, D, E, F, G).

Given that shFOXP1 Th9 CAR‐T cells exhibited superior effector function and resistance to exhaustion in vitro, we next evaluated whether these advantages could be sustained in vivo. A subcutaneous B16‐CD19 melanoma model was established in C57BL/6 mice, followed by adoptive transfer of CAR‐T cells (Figure 5A). Notably, all mice maintained stable body weight, indicating a favorable safety profile of the therapy (Figure S4A). shFOXP1 Th9 CAR‐T treatment markedly suppressed tumor growth compared with Th9 CAR‐T controls (Figure 5B), accompanied by enhanced expansion and persistence of CAR‐T cells in peripheral blood (Figure 5C).

To determine whether these functional benefits were linked to altered differentiation and exhaustion states in vivo, CAR‐T cells were analyzed from the spleen and tumor‐draining lymph nodes. shFOXP1 groups contained a higher proportion of effector‐memory T cells (CD62L^−^CD44^+^) (Figure 5D) and an increased frequency of Ly108^+^PD‐1^+^ stem‐like subsets (Figure 5E,F), together reflecting a balanced effector‐memory composition. In contrast, the proportion of terminally exhausted Ly108^−^CD69^+^ T cells was markedly reduced in the shFOXP1 group (Figure S4B,C). Moreover, splenic CAR‐T cells from the shFOXP1 group expressed higher levels of TNF‐α (Figure 5G), consistent with augmented cytotoxic potential.

Because Tc9 cells represent the CD8^+^ counterpart of IL‐9‐producing T cells, we next examined whether FOXP1 knockdown exerted similar effects in a CD8^+^ IL‐9‐producing CAR‐T context. This comparison was designed to determine whether the functional impact of FOXP1 knockdown reflects a general regulatory mechanism shared by IL‐9‐producing CAR‐T cells, or whether it is preferentially dependent on the CD4^+^ Th9 lineage. Interestingly, although FOXP1 knockdown also promoted polarization in Tc9 CAR‐T cells (Figure S4D,E), these cells exhibited reduced tumor control compared with control Tc9 CAR‐T cells (Figure S4F), indicating that FOXP1 exerts distinct lineage‐specific roles in Th9 vs. Tc9 CAR‐T cells.

In summary, FOXP1 knockdown endows Th9 CAR‐T cells with enhanced persistence, effector‐memory differentiation, and resistance to exhaustion, collectively underpinning their superior antitumor efficacy in vivo.

### 2.6 FOXP1 Knockdown in Th9 CAR‐T Cells Remodels the Tumor Immune Landscape by Recruiting DCs and Expanding Endogenous CD8^+^ T Cells

**FIGURE 6:**
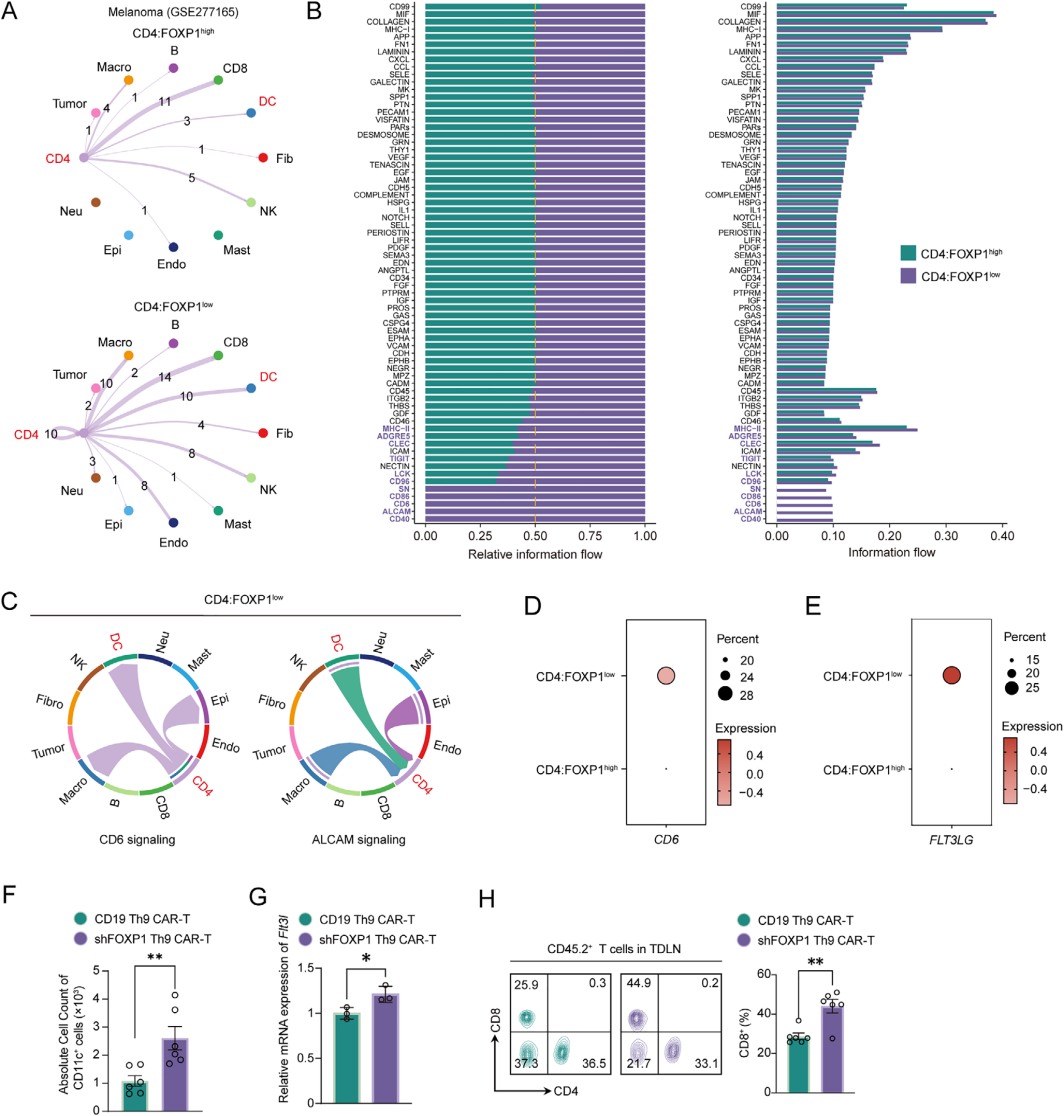
shFOXP1 Th9 CAR‐T cells potentiate endogenous CD8^+^ T cell immunity by recruiting DCs. (A‐E) Single‐cell RNA‐seq analysis from the GSE277165 dataset, including 108,198 cells. These are descriptive cell‐level visualizations without pseudobulk aggregation. (A) Cell‐cell communication interactions of FOXP1‐high CD4^+^ T cells (top) and FOXP1‐low CD4^+^ T cells (bottom) with other cell types, represented as the number of significant ligand‐receptor pairs. (B) Comparison of the number of signaling pathways between FOXP1‐high and FOXP1‐low CD4^+^ T cells. (C) CD6 and ALCAM signaling networks in FOXP1‐low CD4^+^ T cells. (D‐E) Dot plot showing *CD6* (D) and *FLT3LG* (E) expression in FOXP1‐high and FOXP1‐low CD4^+^ T cells. (F) Absolute counts of CD11c^+^ cells in tumor‐draining lymph nodes. Mean ± SEM, n = 6. (G) Expression of *Flt3l* in CD19 Th9 CAR‐T and shFOXP1 Th9 CAR‐T cells. Mean ± SEM, n = 3. (H) Proportions of endogenous CD8^+^ T cells in tumor‐draining lymph nodes. Mean ± SEM, n = 6. *p < 0.05; **p < 0.01; two‐tailed unpaired t test (F, G, H).

Building on the sustained in vivo efficacy of shFOXP1 Th9 CAR‐T cells, we next examined whether their enhanced persistence was associated with remodeling of the endogenous immune landscape. To explore how FOXP1 expression affects intercellular communication within the tumor microenvironment (TME), we constructed a cell–cell interaction network based on published scRNA‐seq datasets from melanoma [40]. Compared with FOXP1‐high CD4^+^ T cells, FOXP1‐low CD4^+^ T cells engaged in more frequent interactions with immune and stromal cell types, particularly dendritic cells (DCs) (Figure 6A). Pathway‐level analysis revealed stronger information flow through the CD40, ALCAM, and CD6 signaling pathways in the FOXP1‐low group (Figure 6B). Directional analysis further revealed that FOXP1‐low CD4^+^ T cells exhibited enhanced bidirectional communication with DCs via the CD6‐ALCAM ligand‐receptor interaction (Figure 6C). This ligand‐receptor pair not only stabilizes DC‐T cell contacts and provides costimulatory signals for T cell activation, but also enables reciprocal signaling whereby activated T cells promote dendritic‐cell maturation and antigen‐presenting capacity [41, 42]. Dot plot analyses consistently confirmed higher *CD6* and *FLT3LG* expression in FOXP1‐low CD4^+^ T cells (Figure 6D,E). Together, these data identify the CD6‐ALCAM interaction as a key communication axis through which FOXP1‐low CD4^+^ T cells may promote DC activation and recruitment.

Consistently, mice treated with shFOXP1 Th9 CAR‐T cells displayed a marked increase in DC numbers compared with controls in tumor‐draining lymph nodes (TDLNs) following CAR‐T therapy (Figure 6F). In parallel, shFOXP1 groups expressed higher levels of *Flt3l* gene, a cytokine essential for DC maturation and proliferation (Figure 6G). Functionally, this immune remodeling was accompanied by a significant expansion of endogenous (CD45.2^+^CD45.1^−^) CD8^+^ T cells in TDLNs (Figure 6H). Meanwhile, analysis of endogenous T cells revealed that they maintained a higher proportion of Ly108^+^PD‐1^+^ stem‐like subsets (Figure S5A–D).

Collectively, these findings indicate that FOXP1 knockdown in Th9 CAR‐T cells enhances cross‐talk with DCs through the CD6‐ALCAM axis and increased Flt3L production, thereby promoting DC recruitment, activation, and endogenous CD8^+^ T cell clonal expansion. The cooperative mechanism may offer a promising approach to control ALVs by engaging the host immune system.

### 2.7 FOXP1 Knockdown Endows Th9 CAR‐T Cells with the Capacity to Eliminate Antigen‐Loss Tumors

**FIGURE 7:**
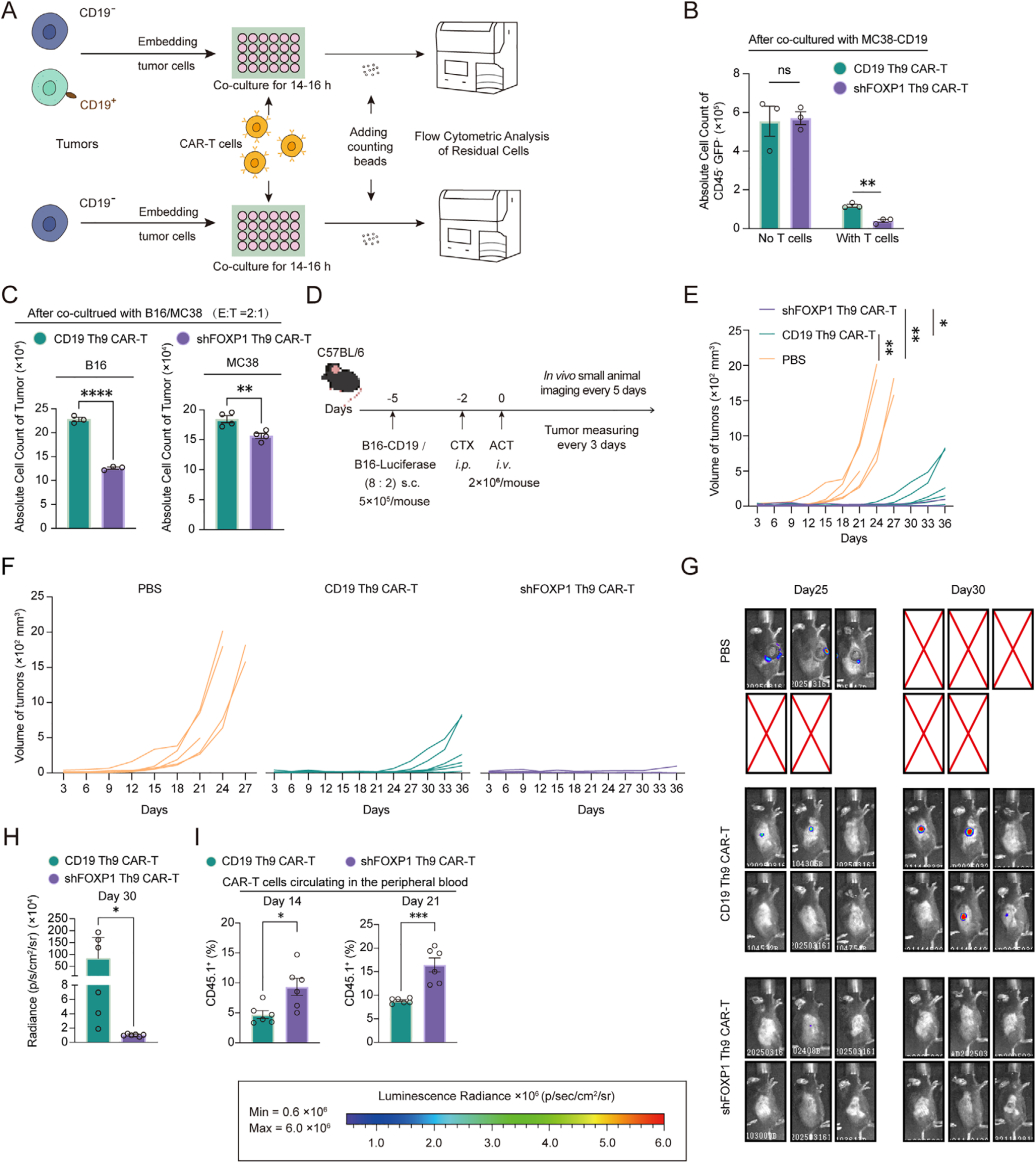
shFOXP1 Th9 CAR‐T therapy exhibits superior efficacy against antigen‐loss tumors. (A) Schematic of the in vitro co‐culture system showing CAR‐T cells incubated with tumor cells lacking the target antigen or with a mixture of antigen‐positive and antigen‐negative tumor cells (blue, MC38 or B16; mint green, MC38‐CD19). (B‐C) Remaining antigen‐negative tumor cells after co‐culture with CAR‐T cells: (B) MC38‐CD19 and MC38 (n = 3) and (C) B16 (n = 3) and MC38 (n = 4). Mean ± SEM. (D) Schematic of the in vivo experimental design. C57BL/6 mice were subcutaneously injected with 5 × 10^5^ B16‐CD19 and B16‐luciferase tumor cells mixed at an 8:2 ratio. Five days after tumor inoculation, cyclophosphamide (CTX) was administered intraperitoneally to deplete endogenous immune cells. Within 48 h of CTX treatment, mice were randomized into three groups and treatment groups were infused intravenously with 2 × 10^6^ Th9 CAR‐T cells respectively. Tumor size was measured every 3 days, and in vivo bioluminescence imaging was performed every 5 days. (E‐F) Tumor growth curves. Mean ± SEM, group PBS, n = 5; group CD19 Th9 CAR‐T, n = 6; group shFOXP1 Th9 CAR‐T, n = 6. (G‐H) Tumor burden assessed by bioluminescence imaging: representative images on days 25 and 30 (G), and quantification of luminescence intensity (H). (I) Frequency of infused CAR‐T cells in peripheral blood. Mean ± SEM, n = 6. *p < 0.05; **p < 0.01; ***p < 0.001; ****p < 0.0001, two‐tailed unpaired t test (B, C, E, H, I).

Having established that shFOXP1 Th9 CAR‐T cells remodel the immune landscape and enhance endogenous CD8^+^ T cell responses, we next examined whether these cells could directly target and eliminate ALVs. To this end, we first evaluated their cytotoxicity against tumor cells lacking the CD19 antigen using an in vitro killing assay (Figure 7A). The shFOXP1 groups exhibited markedly stronger killing of both B16 and MC38 tumor cells, irrespective of CD19 expression, indicating enhanced cytotoxic activity beyond target‐antigen recognition (Figure 7B,C).

We then established a mixed‐antigen ALV tumor model by co‐injecting CD19^+^ (B16‐CD19) and CD19^−^ (B16‐luciferase) tumor cells with an 8:2 ratio subcutaneously, followed by adoptive transfer of CAR‐T cells (Figure 7D). Consistent with their in vitro potency, mice treated with shFOXP1 Th9 CAR‐T cells showed significantly delayed tumor growth compared with controls (Figure 7E,F), and bioluminescence imaging further confirmed superior tumor clearance in the shFOXP1 group (Figure 7G,H). Moreover, a higher frequency of circulating CAR‐T cells was detected in peripheral blood (Figure 7I), indicating improved persistence of shFOXP1 Th9 CAR‐T cells even within the ALV system.

Together, these results demonstrate that FOXP1 knockdown endows Th9 CAR‐T cells with dual antitumor capacity, enabling effective elimination of both antigen‐positive and antigen‐loss tumor populations. This property underscores their potential to overcome immune escape and achieve durable tumor control.

### 2.8 FOXP1 Knockdown Potentiates Th9 CAR‐T Cell Effector Function Through Coordinated Regulation of Signaling Pathways and Effector Programs

**FIGURE 8:**
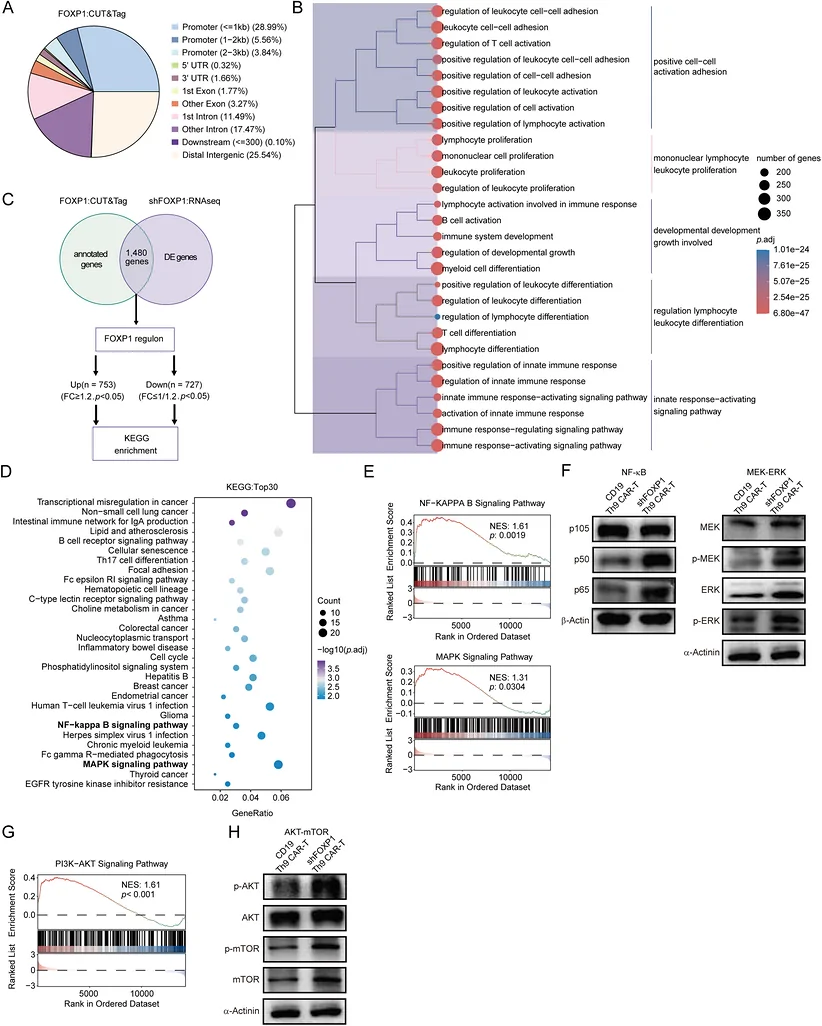
FOXP1 knockdown enhances Th9 CAR‐T cell effector function through coordinated regulation of multiple signaling pathways. (A) Pie chart showing the genomic distribution of FOXP1 CUT&Tag peaks in CD4^+^ T cells. (B) GO enrichment analysis of FOXP1‐bound genes displayed as a tree plot. (C) Integration of FOXP1 CUT&Tag‐annotated genes with differentially expressed (DE) genes from shFOXP1 Th9 CAR‐T vs. CD19 Th9 CAR‐T bulk RNA‐seq identified 1,480 candidates (the FOXP1 regulon). The regulon was divided into upregulated (n = 753; fold change ≥ 1.2, *p* < 0.05) and downregulated (n = 727; fold change ≤ 1/1.2, *p* < 0.05) subsets, which were subjected to KEGG pathway enrichment analyses. (D) Bubble plot showing the top 30 significantly enriched KEGG pathways (adjusted *p* < 0.05) among the upregulated gene set. (E) GSEA of bulk RNA‐seq comparing shFOXP1 Th9 CAR‐T with CD19 Th9 CAR‐T showing enrichment of the NF‐κB signaling pathway (top) and the MAPK signaling pathway (bottom). (F) Western blot analysis of total NF‐κB components (p105, p50, p65), total and phosphorylated MAPK components (MEK, ERK). (G) GSEA of bulk RNA‐seq comparing shFOXP1 Th9 CAR‐T with CD19 Th9 CAR‐T showing enrichment of the PI3K‐AKT signaling pathway. (H) Western blot analysis of total and phosphorylated AKT and mTOR proteins.

To investigate the molecular mechanisms underlying the enhanced antitumor activity of shFOXP1 Th9 CAR‐T cells, we performed FOXP1 CUT&Tag sequencing and peak annotation in Th9 cells. As a transcription factor, FOXP1 exhibited binding enrichment primarily at proximal promoters (≤1 kb) and distal intergenic regions (Figure 8A), consistent with its role in promoter and enhancer regulation. GO enrichment analysis of FOXP1‐bound genes revealed associations with key biological processes, including T cell activation, differentiation, immune response, and cell adhesion (Figure 8B).

Analysis of FOXP1 CUT&Tag profiles further revealed binding peaks at the *Il9* locus and multiple transcription factors related to Th9 differentiation (*Spi1*, *Bach2*, *Runx1*), as well as effector genes (*Gzmb*, *Tnf*) and TCR signaling components (*Zap70*, *Lck*, *Rela*) (Figure S6A–C) [43]. These findings suggest that FOXP1 exerts broad transcriptional control over both lineage‐defining and effector programs.

To further identify pathways directly influenced by FOXP1 loss, we intersected CUT&Tag‐annotated genes with significantly differentially expressed genes from shFOXP1 vs. control Th9 CAR‐T RNA‐seq, yielding 1,480 overlapping targets (Figure 8C). KEGG pathway analysis of upregulated genes revealed strong enrichment of NF‐κB, MAPK, and PI3K‐Akt signaling pathways (Figure 8D, Table S3). GSEA and protein validation confirmed these findings: NF‐κB and MAPK pathways were transcriptionally enriched in shFOXP1 Th9 CAR‐T cells (Figure 8E), accompanied by increased phosphorylation of corresponding signaling molecules (Figure 8F). Similarly, PI3K‐Akt/mTOR signaling was significantly activated at both transcript and protein levels (Figure 8G,H).

Beyond these core pathways, transcriptomic GSEA indicated additional upregulation of AGE‐RAGE, cGMP‐PKG, JAK‐STAT, and Ca^2^^+^‐related signaling (Figure S6D). Importantly, pseudobulk GSEA of both mouse and human single‐cell datasets revealed consistent enrichment of TCR‐associated signaling pathways in FOXP1‐low CD4^+^ T cells (Figure S6E,F) [38, 39], indicating a conserved regulatory pattern across species.

Together, these results suggest that FOXP1 knockdown relieves transcriptional repression of IL‐9‐related and effector genes, while concurrently enhancing TCR downstream signaling pathways (MAPK, NF‐κB, PI3K‐Akt etc.), thereby synergistically amplifying the effector function and antitumor activity of Th9 CAR‐T cells.

## 3 Discussion

CAR‐T cell therapy has transformed cancer treatment but remains limited by antigen‐loss‐mediated relapse, particularly in solid tumors where target heterogeneity and immune suppression prevail [15]. In this study, we identify FOXP1 as a key transcriptional repressor restraining Th9 CAR‐T differentiation and effector programming. FOXP1 knockdown reprogrammed Th9 CAR‐T cells toward a more cytotoxic, metabolically active, and exhaustion‐resistant phenotype, thereby markedly enhancing antitumor efficacy. Beyond direct tumor clearance, FOXP1‐deficient Th9 CAR‐T cells promoted DC recruitment and endogenous CD8^+^ T cell activation, enabling effective elimination of ALVs that typically escape conventional CAR‐T therapy. These findings reveal FOXP1 as a central transcriptional checkpoint controlling Th9 lineage fate and establish a mechanistic framework for engineering CAR‐T cells capable of overcoming antigen escape.

FOXP1 has long been recognized as a transcriptional brake that maintains T cell quiescence and limits effector activation [25, 44]. It directly suppresses *Il7r* to restrain IL‐7‐PI3K‐Akt‐mTOR signaling and represses lineage‐defining and effector genes such as *Il21*, *Icos*, *Gzmb*, and *Ifng*, thereby preventing premature activation and differentiation [27, 45]. In Th9 cells, we have reported that FOXP1 binds to the *Il9* promoter and antagonizes the FOXO1‐P300 complex, which otherwise promotes *Il9* transcription [25]. Consistent with these prior findings, our CUT&Tag and RNA‐seq analyses revealed FOXP1 occupancy at the promoters and enhancers of *Il9*, *Spi1*, and *Runx1*, as well as effector loci including *Tnf* and *Gzmb*, confirming its role as a global repressor of both Th9‐lineage and cytotoxic programs. FOXP1 knockdown relieved this repression and reactivated multiple signaling pathways including NF‐κB, MAPK, and PI3K‐Akt/mTOR that govern effector differentiation and metabolic reprogramming. This coordinated activation was accompanied by increased IL‐9 and TNF‐α production and enhanced effector‐memory differentiation, suggesting that FOXP1 simultaneously constrains cytokine and TCR‐downstream signaling programs. Its suppression thus enables synchronized transcriptional and metabolic activation, endowing Th9 CAR‐T cells with superior persistence and functional durability. However, whether complete FOXP1 knockout would further improve the therapeutic efficacy of Th9 CAR‐T cells remains unknown and warrants systematic investigation in future studies.

The lineage‐dependent effect of FOXP1 knockdown was further supported by our Tc9 CAR‐T data. Tc9 cells are IL‐9‐producing CD8^+^ T cells and therefore serve as a CD8^+^ counterpart to CD4^+^ Th9 cells. Although FOXP1 knockdown increased IL‐9‐associated polarization in Tc9 CAR‐T cells, it reduced their therapeutic efficacy, in contrast to the enhanced antitumor activity observed in shFOXP1 Th9 CAR‐T cells. Interestingly, a recent study reported that FOXP1 has been shown to exert a dual role in CD8^+^ CAR‐T cells. FOXP1 deletion initially enhanced CD8^+^ CAR‐T activation but ultimately impaired tumor control by accelerating terminal differentiation and exhaustion of T cells [43]. Similarly, we found that FOXP1 knockdown increased IL‐9 production in Tc9 CAR‐T cells yet reduced their therapeutic efficacy, highlighting FOXP1's context‐dependent role in balancing activation and persistence. In contrast, the Th9 lineage appears uniquely equipped to buffer excessive activation through IL‐9‐driven autocrine and paracrine feedback circuits that sustain survival and limit premature exhaustion [21]. CD8^+^ T cells, although highly cytotoxic, depend on CD4^+^ T cell help for sustained function and readily undergo TOX^+^PD‐1^+^TIM‐3^+^ exhaustion under chronic antigen stimulation [46, 47, 48]. By contrast, CD4^+^ T cells act as immunoregulatory hubs, exhibiting superior persistence, metabolic flexibility, and the capacity to sustain CD8^+^ T cell effector activity through cytokine support and microenvironmental remodeling [49, 50, 51]. These differences explain why FOXP1 knockdown confers a net functional advantage in Th9 CAR‐T cells but not in CD8^+^ counterparts, and they suggest that CD4^+^ rather than bulk CD3^+^ or CD8^+^ T cells may represent a more suitable backbone for clinical translation of shFOXP1‐engineered CAR‐T therapy. Thus, the Tc9 comparison helps define the mechanistic boundary and lineage‐specific applicability of the shFOXP1 Th9 CAR‐T strategy. Notably, FOXP1 has also been reported to positively regulate aspects of CD8^+^ T cell homeostasis and memory maintenance [43], further supporting the notion that its functional outcomes are lineage‐specific and context‐dependent.

ALVs remain a major obstacle to durable tumor control after adoptive cell therapy [13]. Conventional approaches, such as dual, tandem, or sequential CAR designs and universal adapter systems, aim to broaden antigen recognition but do not fundamentally address the immunological consequences of antigen escape [52, 53]. An emerging alternative is to amplify bystander or systemic antitumor immunity that extends beyond target specificity [54]. Among T‐helper subsets, Th9 cells are uniquely capable of eliminating ALVs through a “viral‐mimicry” mechanism: the absence of CD39 in Th9 cells causes extracellular ATP accumulation, activating the TLR3‐MAVS‐type I interferon axis in tumor‐infiltrating myeloid cells, leading to DC activation, IFN‐α/β release, and recruitment of endogenous CD8^+^ T cells [19]. Consistent with this model, our study demonstrates that FOXP1 knockdown reinforces this circuit. Mechanistically, FOXP1 knockdown amplifies the intrinsic advantages of Th9 CAR‐T cells against ALVs through two complementary mechanisms: (i) relieving FOXP1‐mediated transcriptional repression to enhance IL‐9 secretion and effector differentiation, thereby driving DC recruitment and endogenous CD8^+^ T cell priming; and (ii) shifting the effector‐exhaustion balance toward a persistent, functional state. In mixed‐antigen tumors containing both CD19^+^ and CD19^−^ cells, shFOXP1 Th9 CAR‐T therapy achieved sustained tumor control accompanied by increased DC infiltration and elevated *Flt3l* expression, which supports DC maturation and expansion. This remodeling of the tumor immune landscape was associated with robust clonal expansion of endogenous CD8^+^ T cells, indicating that FOXP1‐deficient Th9 CAR‐T cells not only enhance direct cytotoxicity but also mobilize host antitumor immunity to eradicate antigen‐deficient clones. However, we also observed that the mixed CD19^+^/CD19^−^ ALV tumor model exhibited a slower growth rate than the conventional CD19^+^ tumor model, even in the absence of therapeutic treatment. Therefore, differences in tumor composition, mixing ratio, or tumor model may influence the apparent efficacy of antigen‐loss tumor eradication. This limitation should be considered when interpreting the therapeutic magnitude of FOXP1‐suppressed Th9 CAR‐T cells in the ALV setting.

Collectively, these findings support a broader conceptual shift in addressing antigen escape: durable ALV control requires integration of direct cytolytic activity with immune network amplification. FOXP1 knockdown in Th9 CAR‐T cells achieves this balance by inducing a broad yet calibrated activation of TCR‐downstream pathways that strengthen effector programs while restraining exhaustion and promoting memory formation. Unlike single‐pathway interventions that are readily suppressed by inhibitory cues such as TGF‐β or PD‐L1, FOXP1 modulation establishes a redundant, self‐sustaining signaling network that maintains Th9 CAR‐T function even under immunosuppressive pressure. This broad‐spectrum, lineage‐tuned activation provides a mechanistic rationale for the superior durability of FOXP1‐deficient Th9 CAR‐T cells and offers a translatable strategy to overcome tumor antigen heterogeneity. Future studies should explore pharmacologic or inducible modulation of FOXP1 to fine‐tune CAR‐T activation and assess potential synergy with immune checkpoint blockade or metabolic reprogramming, ultimately aiming to achieve durable, antigen‐independent tumor eradication.

## 4 Methods

### 4.1 scRNA‐seq Data Processing and Clustering

Single‐cell transcriptomic data were processed using Seurat (v4.4.0) [55]. Gene expression matrices were normalized with the NormalizeData function, and the top 2,000 highly variable genes were identified using FindVariableFeatures. Data were scaled with ScaleData, followed by principal component analysis (PCA) using RunPCA under default Seurat settings. Subsequently, batch effects were corrected on the PCA embeddings using RunHarmony [56], with patient identity specified as the grouping variable. Clustering was performed by shared nearest neighbor graph construction using FindNeighbors (reduction = “harmony”, dims = 1:10), followed by graph‐based clustering with FindClusters (resolution = 0.4), with default values used for all other parameters. UMAP visualization was generated using RunUMAP (reduction = “harmony”, dims = 1:10). T cell subsets were annotated based on canonical marker gene expression as follows:

#### 4.1.1 CD4^+^ T Cells

Tnaive (*CCR7, IL7R*), Tcm (*TCF7, RGS1*), Tem (*GZMA, CCL5*), Teff (*KLRG1, CX3CR1*), Tex (*PDCD1, LAG3*), Treg (*FOXP3, CCR8*);

#### 4.1.2 CD8^+^ T Cells

Tnaive (CCR7, LEF1), Tcm (IL7R, PRF1), Tem (GZMK, CXCR4), Trm (CD6, XCL1), Teff (KLRG1, CX3CR1), Tex (PDCD1, HAVCR2), Prolif (MKI67, STMN1).

FOXP1 expression levels across subsets were visualized using DotPlot and VlnPlot. Gene expression density maps were generated using Nebulosa (v1.16.0) [57].

### 4.2 Bulk RNA‐seq Analysis

Th9 CAR‐T and control CAR‐T cells were cultured in vitro for 5 days, and total RNA was extracted using the AG RNAex Pro kit (AG21102). RNA quality was assessed by Biomarker Technologies. Libraries were prepared and sequenced on an Illumina NovaSeq 6000 platform. Raw reads were trimmed with Trim Galore to remove adapters and low‐quality sequences, then aligned to the mouse genome (GRCm38) using HISAT2. Gene‐level counts were obtained using featureCounts, and expression was normalized to FPKM (fragments per kilobase of exon per million mapped reads). Differentially expressed genes were identified using DESeq2 (v1.44.0) [58] with adjusted *p* < 0.05 and |log2 fold change| ≥ log2(1.2). Volcano plots were generated with ggplot2 (v3.5.1), and heatmaps were visualized using pheatmap (v1.0.12) with row‐wise normalization.

### 4.3 Pseudobulk RNA‐seq Analysis

Pseudobulk RNA‐seq analysis was performed to identify differentially expressed genes between the FOXP1‐low and FOXP1‐high groups in CD4^+^ T cells. The average FOXP1 expression in CD4^+^ T cells was calculated for each sample. For the human data, this was defined at the patient level, whereas for the mouse data, samples from the GSE152022 and GSE298152 datasets were additionally included because of the limited sample size in the GSE240762 dataset. Samples were then classified into FOXP1‐high and FOXP1‐low groups using the median average FOXP1 expression across samples as the cutoff. Raw transcript counts were subsequently aggregated by sample using AggregateExpression in Seurat to construct a pseudo‐bulk expression matrix. Differential gene expression analysis between the FOXP1‐low and FOXP1‐high groups was performed using DESeq2 (v1.44.0).

### 4.4 Pseudotime Analysis

Pseudotime analysis was performed using Monocle2 (v2.26.0) to investigate the evolutionary trajectories of CD4^+^ and CD8^+^ T‐cell populations [59]. Cells were stratified by cell type and subsampled from the total T‐cell population, with 2000–2500 cells retained for each cell type, and a CellDataSet object was constructed based on the raw count matrix. After estimating size factors and dispersion, ordering genes were selected using differentialGeneTest. Dimensionality reduction was then performed using DDRTree, followed by cell ordering with orderCells to infer pseudotime trajectory. Cell trajectory distribution and the dynamic expression pattern of FOXP1 along pseudotime were visualized using plot_cell_trajectory and plot_genes_in_pseudotime, respectively. Genes significantly associated with pseudotime were further identified using differentialGeneTest.

### 4.5 Definition of the FOXP1^dim^ CD4^+^ T Cell and Th9 Signatures

Based on the GSE156728 scRNA‐seq dataset, patients were divided into FOXP1‐high and FOXP1‐low groups according to the median average FOXP1 expression in CD4^+^ T cells across all patients. Pseudo‐bulk differential expression analysis was then performed using DESeq2 (v1.44.0), and significantly upregulated genes in the FOXP1‐low group (adjusted *p* < 0.05, log2 fold change ≥ 1.0) were selected to define the FOXP1^dim^ CD4^+^ T‐cell signature (Table S1).

For the Th9 signature, RNA‐seq count data for Th9 and Th1 cells were obtained from GEO (GSE156075). Differential expression analysis with DESeq2 (v1.44.0) identified 1,048 genes significantly upregulated in Th9 vs. Th1 cells (adjusted *p* < 0.05, log2 fold change ≥ 1.0), which were used to characterize Th9‐related signaling pathways. The top 100 genes ranked by fold change were selected as the Th9 signature (Table S1).

### 4.6 Signature Scoring and Sample‐ or Patient‐Level Analysis of scRNA‐seq Data

Th9, cytotoxicity, and activation‐effector‐function signature scores were calculated at the single‐cell level using Seurat's AddModuleScore function with predefined gene sets (Table S1). FOXP1 expression was obtained from the normalized RNA expression matrix. Within each sample or patient, cells with FOXP1 expression above the sample‐ or patient‐specific median were classified as FOXP1‐high, whereas the remaining cells were classified as FOXP1‐low. For each signature, the mean score was calculated separately for FOXP1‐high and FOXP1‐low cells within each sample or patient. Paired sample‐ or patient‐level scores were compared using a two‐sided Wilcoxon signed‐rank test, with samples or patients treated as the independent units of statistical analysis.

### 4.7 Survival Analysis

Overall survival analysis for the TCGA pan‐cancer cohort was performed using the GEPIA2 website [60]. Tumor types with more than 200 samples were included in the analysis, including BLCA, BRCA, CESC, COAD, HNSC, KIRC, KIRP, LIHC, LUAD, LUSC, OV, PRAD, SARC, SKCM, STAD, and THCA. Patients were divided into high‐ and low‐score groups using the median signature score as the cutoff. For the chronic lymphocytic leukemia cohort, Kaplan–Meier survival curves were generated using the survival (v3.6.4) and survminer (v0.4.9) packages, with the optimal cut‐point used for group stratification.

### 4.8 TIMER2.0‐Based Immune Infiltration Analysis

Immune infiltration analysis was performed using the Immune Association module of the TIMER2.0 web server [37]. The association between FOXP1 expression and immune cell‐type abundance across TCGA tumor types was analyzed using the TIMER2.0 deconvolution framework, including CD4^+^ T cells, CD8^+^ T cells, Tregs, NK cells, NKT cells, macrophages, cancer‐associated fibroblasts, and dendritic cells. Correlation results were retrieved from TIMER2.0 (Table S2), and heatmaps were generated using ggplot2(v3.5.1) in R.

### 4.9 Cell‐Cell Communication Analysis

Melanoma single‐cell data are available from GEO (GSE277165). Within the CD4^+^ T cell populations, cells were stratified into FOXP1‐high and FOXP1‐low groups based on standardized FOXP1 expression using a 30th/70th percentile split. Cell‐cell communication networks were inferred using the CellChat package (v1.6.1) [61], and the number of significant ligand‐receptor interactions between cell clusters was quantified for each group. Comparative analysis of signaling pathway activity between the two groups was performed using rankNet, and the overall information flow of the inferred signaling networks was visualized. Chord diagrams were generated using netVisual_chord_cell to display the CD6 and ALCAM signaling interactions in the FOXP1‐low group.

### 4.10 Gene Set Enrichment Analysis

GSEA was performed using clusterProfiler (v4.12.0) [62] and visualized with GseaVis (v0.0.2). Gene sets were derived from the Gene Ontology (GO), Kyoto Encyclopedia of Genes and Genomes (KEGG), and MSigDB Hallmark gene set collections.

### 4.11 CUT&Tag Assay

CUT&Tag assays were performed using the Cut&Tag Kit (Novoprotein, N259‐YH01) according to the manufacturer's protocol. Briefly, cell suspensions were incubated with ConA‐coated magnetic beads for 10 min at room temperature, followed by magnetic separation and incubation with target‐specific primary antibodies for 2 h. After washing, samples were incubated with secondary antibodies for 1 h and treated with ChiTag transposase complexes to initiate tagmentation in Tagmentation Buffer (10 min, RT). Reactions were terminated, and DNA was purified using Tagment DNA Extract Beads. Purified DNA was PCR‐amplified to generate sequencing libraries.

Raw reads were quality‐assessed with FastQC and aligned to the mouse genome (GRCm38) using Bowtie2. Alignments were converted (SAM→BAM) and sorted with samtools, followed by transcription factor peak calling with MACS2. Peaks were annotated using ChIPseeker (v1.40.0) [63], and the genomic distribution of FOXP1 CUT&Tag peaks was visualized using the plotAnnoPie function. Downstream functional enrichment of GO terms and KEGG pathways was performed with clusterProfiler (v4.12.0). Transcription factor binding profiles were visualized in IGV (Integrated Genomics Viewer).

### 4.12 Experimental Animals and Cell Lines

C57BL/6 mice were obtained from the Laboratory Animal Center of Southern Medical University and the Guangdong Medical Laboratory Animal Center. CD45.1 (B6.SJL‐Ptprca Pepcb/BoyJ) mice were provided by Professor Bing Sun.

All animal experiments were approved by the Institutional Animal Care and Use Committee of Southern Medical University, Guangzhou, Guangdong, China (approval No. SMUL202502017). And experiments were performed in accordance with institutional guidelines and the approved experimental animal research protocol.

B16, B16‐CD19, B16‐Luciferase, MC38, MC38‐CD19, and 293T cell lines were maintained in Dulbecco's modified Eagle medium (DMEM; Biological Industries) supplemented with 10% heat‐inactivated fetal bovine serum (FBS; Gibco) and 100 U/mL penicillin‐streptomycin (Gibco).

### 4.13 Western Blotting

Cells were lysed on ice in 1× lysis buffer (NCM, WB3100) supplemented with a protease inhibitor cocktail (Solarbio, P1260). Protein concentrations were quantified, and 10–20 µg of protein per sample was separated by 10% SDS‐PAGE (Fisher) and transferred onto PVDF membranes via standard wet transfer. Membranes were blocked with 5% nonfat milk in TBST and incubated overnight at 4 °C with primary antibodies, followed by HRP‐conjugated secondary antibodies. Signals were detected by enhanced chemiluminescence (ECL).

### 4.14 Quantitative Real‐Time PCR (qPCR)

Total RNA was extracted from T cells using RNAex Pro reagent (AG, AG21102). cDNA was synthesized with the Hifair III Reverse Transcriptase Master Mix (YEASEN, 11137ES10), and qPCR was performed on a QuantStudio 3 Real‐Time PCR System (Thermo Fisher Scientific) using ChamQ Universal SYBR qPCR Master Mix (Vazyme, Q711‐02). Primers were synthesized by Ruibiotech. Gene expression levels were normalized to β‐actin as an internal reference.

### 4.15 T Cell Isolation, Activation, and Co‐Culture

Naïve CD4^+^ and CD8^+^ T cells were isolated from spleens of C57BL/6 or CD45.1 mice using MojoSort Naïve CD4^+^/CD8^+^ T Cell Isolation Kits (BioLegend, 480040/480035). Cells were cultured in RPMI‐1640 medium supplemented with 10% FBS, 0.1% β‐mercaptoethanol, 1% L‐glutamine, and 1% penicillin‐streptomycin at 5 × 10^5^ cells per well in 48‐well plates.

For Th9 differentiation, naïve CD4^+^ T cells were activated with plate‐bound anti‐CD3 antibody (5 µg/mL; Bio X Cell, clone 17A2) and soluble anti‐CD28 antibody (1 µg/mL; Bio X Cell, clone 37.51) under Th9‐polarizing conditions containing IL‐4 (10 ng/mL; Novoprotein, CK74), TGF‐β (1 ng/mL; Novoprotein, GMP‐CA59), and anti‐IFN‐γ neutralizing antibody (20 µg/mL; clone XMG1.2). After 3 days of culture, cells were collected and further maintained in T‐cell medium supplemented with IL‐4 and TGF‐β.

For Tc9 differentiation, naïve CD8^+^ T cells were activated under conditions identical to those used for Th9 differentiation, with the addition of recombinant IL‐2 (100 U/mL; Novoprotein, C013).

For cytotoxicity assays, mixed B16‐CD19 with B16 (Ratio 1:1) or mixed MC38‐CD19 with MC38 (Ratio 1:1) tumor cells were co‐cultured with preactivated T cells at effector‐to‐target (E:T) ratios of 0:1, 1:1, or 4:1 in 48‐well plates. For antigen‐negative bystander cytotoxicity assays, B16 or MC38 tumor cells were co‐cultured with preactivated T cells at an effector‐to‐target (E:T) ratio of 2:1 in 48‐well plates. After 16 h, cells were collected for flow cytometry. Tumor cells were identified as CD45^−^GFP^+^ for cytotoxicity quantification. In antigen‐loss models, target‐negative tumor cells (CD45^−^GFP^−^) were used to evaluate bystander killing efficiency.

### 4.16 LDH assay

CAR‐T cell‐mediated cytotoxicity was further evaluated using the LDH Cytotoxicity Assay Kit (Beyotime, C0017) according to the manufacturer's instructions. Briefly, B16‐CD19 target tumor cells were seeded in 48‐well plates and co‐cultured with CD19 Th9 CAR‐T or shFOXP1 Th9 CAR‐T cells at the indicated effector‐to‐target (E:T) ratios of 1:1, 2:1, and 3:1. After 8, 16, or 24 h of co‐culture, plates were centrifuged to pellet cells, and the culture supernatants were collected for LDH measurement. Spontaneous LDH release was determined from target cells cultured without effector cells, whereas maximum LDH release was determined from target cells treated with lysis buffer. Absorbance was measured using a microplate reader, and cytotoxicity was calculated as follows: cytotoxicity (%) = [(experimental LDH release—T cell spontaneous LDH release) / (maximum LDH release—tumor spontaneous LDH release)] × 100.

### 4.17 Flow Cytometry

Cells from in vitro and in vivo experiments were stained with fluorochrome‐conjugated antibodies. Viability staining was performed in PBS for 5 min in the dark at room temperature. Surface staining was performed in ice‐cold PBS containing 0.5% BSA for 30 min in the dark, followed by two washes. Intracellular cytokines were detected using the BD Intracellular Staining Kit (BD Biosciences, 554714) according to the manufacturer's instructions. Nuclear transcription factors were stained using the eBioscience Foxp3/Transcription Factor Staining Buffer Set (Thermo Fisher Scientific, 00‐5523‐00). Samples were acquired on BD LSRFortessa X‐20, BD FACSCanto II, or Beckman Coulter CytoFLEX LX flow cytometers, and analyzed with FlowJo (v10.8.1; Tree Star).

### 4.18 Antibodies and Reagents for Flow Cytometry and Western Blotting

The following antibodies and staining reagents were used:

#### 4.18.1 From BioLegend

APC anti‐mouse CD4 (SK3, 980812), PE anti‐mouse TNF‐α (MP6‐XT22, 506306), APC anti‐human/mouse Granzyme B (QA16A02, 372204), APC anti‐mouse CD152 (CTLA‐4; UC10‐4B9, 106310), PE anti‐mouse Ly108 (330‐AJ, 134606), Brilliant Violet 421 anti‐mouse PD‐1 (29F.1A12, 135218), APC anti‐mouse PD‐1 (29F.1A12, 135210), PE/Cyanine7 anti‐mouse PD‐1 (29F.1A12, 135216), PE/Cyanine7 anti‐mouse CD69 (H1.2F3, 104512), PerCP/Cyanine5.5 anti‐mouse CD8α (S18018E, 162310), APC anti‐mouse CD8α (S18018E, 162306), Brilliant Violet 785 anti‐mouse CD8α (53‐6.7, 100749), PE anti‐mouse CD11b (M1/70, 101208), Brilliant Violet 605 anti‐mouse CD11c (M1/70, 101237), PE anti‐mouse CD11c (N418, 117308), APC anti‐mouse Granzyme B (QA16A02, 372204), PE/Cyanine7 anti‐mouse CD45 (S18009F, 157206), PE anti‐mouse CD45.1 (A20, 110708), PerCP/Cyanine5.5 anti‐mouse CD45 (30‐F10, 103131), PE/Cyanine7 anti‐mouse CD45.2 (104, 109830), Brilliant Violet 510 anti‐mouse/human CD44 (IM7, 103043), PE/Cyanine7 anti‐mouse/human CD44 (QA19A43, 163608), and PerCP/Cyanine5.5 anti‐mouse CD62L (MEL‐14, 104432).

#### 4.18.2 For Signaling Analysis

anti‐phospho‐AKT (649001), anti‐mTOR (6H9B10, 659202), anti‐STAT5 (9C8B50, 660202), and anti‐phospho‐STAT5 (A17016B, 936902).

#### 4.18.3 From BioXcell

Anti‐IFN‐γ (XMG1.2, BE0055), anti‐mouse CD3 (17A2, BE0002), and anti‐mouse CD28 (37.51, BE0015‐1).

#### 4.18.4 From Cell Signaling Technology

Fixable Viability Dye eFluor 780 (65‐0865‐14), FOXP1 (D35D10, 4402S), MEK1/2 (L38C12, 4694S), and phospho‐MEK1/2 (41G9, 9154).

#### 4.18.5 From BeyoTime

α‐Actinin (O43707, AG1022).

#### 4.18.6 From ImmunoWay

Phospho‐mTOR (PT0498R, YM8326).

#### 4.18.7 From Proteintech

NF‐κB p105/p50 (14220‐1‐AP) and NF‐κB p65 (10745‐1‐AP).

### 4.19 Establishment of Tumor Models and Adoptive Cell Therapy

C57BL/6 mice were subcutaneously inoculated in the right flank with 5 × 10^5^ B16‐CD19 melanoma cells. For adoptive T cell therapy, naive CD4^+^ T cells were isolated from mouse spleens, differentiated under Th9 or Tc9 condition, and transduced with CAR‐encoding retrovirus on 40 h after T cell activation. The cells were then expanded in the presence of IL‐4 and TGF‐β to polarize them into Th9 cells over a total culture period of 5 days. On day 5 post‐inoculation, 2 × 10^6^ in vitro‐differentiated Th9 cells were administered via tail vein injection. Lymphodepletion was induced by intraperitoneal injection of cyclophosphamide (CTX; 200 mg/kg; Sigma–Aldrich, C7397) 24 to 48 h before T cell transfer.

At indicated time points, mice were euthanized for collection of tumor‐derived single‐cell suspensions, splenocytes, and tumor‐draining lymph node (tdLN) cells.

For the antigen‐loss variant (ALV) tumor model, mice were inoculated with an 8:2 mixture of B16‐CD19 and B16‐luciferase cells using the same protocol. Tumor progression was monitored every 5 days via in vivo bioluminescence imaging. Following intraperitoneal injection of D‐luciferin potassium salt (150 mg/kg; APEBIO, C3654), mice were anesthetized with 2% isoflurane/oxygen. Bioluminescent images were acquired on an IVIS Spectrum Imaging System, and tumor burden was quantified as radiance (photons/s/cm^2^/sr) using Living Image software (v4.7.2) by defining regions of interest (ROIs) around tumor sites.

## Author Contributions

E. B. initiated the study, E. B., A. X., W. Z. and H. L. designed the experiments, and wrote the paper; Y. Z. (Yihan Zhu), X. X. and S. O. performed most of the experiments and statistical analyses; X. W. performed all the bioinformatic analysis; Y. Z (Yang Zhou), W. Z., K. W., Y. Z. (Yutong Zhong), Y. C. and L. J. help with the experiments; H. W. and Y. G. helped with experiment design and provided critical suggestions.

## Consent

This study was not a clinical trial. No written consent has been obtained from the patients as there is no patient identifiable data included in this case report/series.

## Conflicts of Interest

The authors declare no conflicts of interest.

## Supporting information




**Supporting File 1**: advs77564‐sup‐0001‐SuppMat.pdf.

**Supporting File 2**: advs77564‐sup‐0002‐SuppMat.xlsx.

## Data Availability

All data supporting the findings of this study are available in the main text and/or Supplemental Materials. The raw RNA‐seq and CUT&Tag data reported in this paper have been deposited in the Genome Sequence Archive (GSA) under accession CRA032152. Published single‐cell datasets related to CAR‐T therapy and immune checkpoint inhibitor cohorts were obtained from GEO or cited references: GPC3‐positive solid tumors (GSE253352), glioma (GSE186802), triple‐negative breast cancer (GSE169246), non‐small cell lung cancer (GSE179994), basal cell carcinoma (GSE123813), and renal cell carcinoma (dataset published in Bi K et al. [35].). For the chronic lymphocytic leukemia cohort, CAR‐stimulated T cell RNA‐seq and survival data were retrieved from Fraietta et al. [32].
