# Supplementary material for: FOXP1 Knockdown Reprograms Th9 CAR‐T Cells to Overcome Antigen Escape
Source: Adv Sci (Weinh). 2026 Sep 16:e77564. Online ahead of print. doi: 10.1002/advs.77564 (PMC13583099; doi:10.1002/advs.77564)
Supplement: Supplementary file 1 [file ADVS-9999-e77564-s002.pdf]

Supplementary Figure Legend:

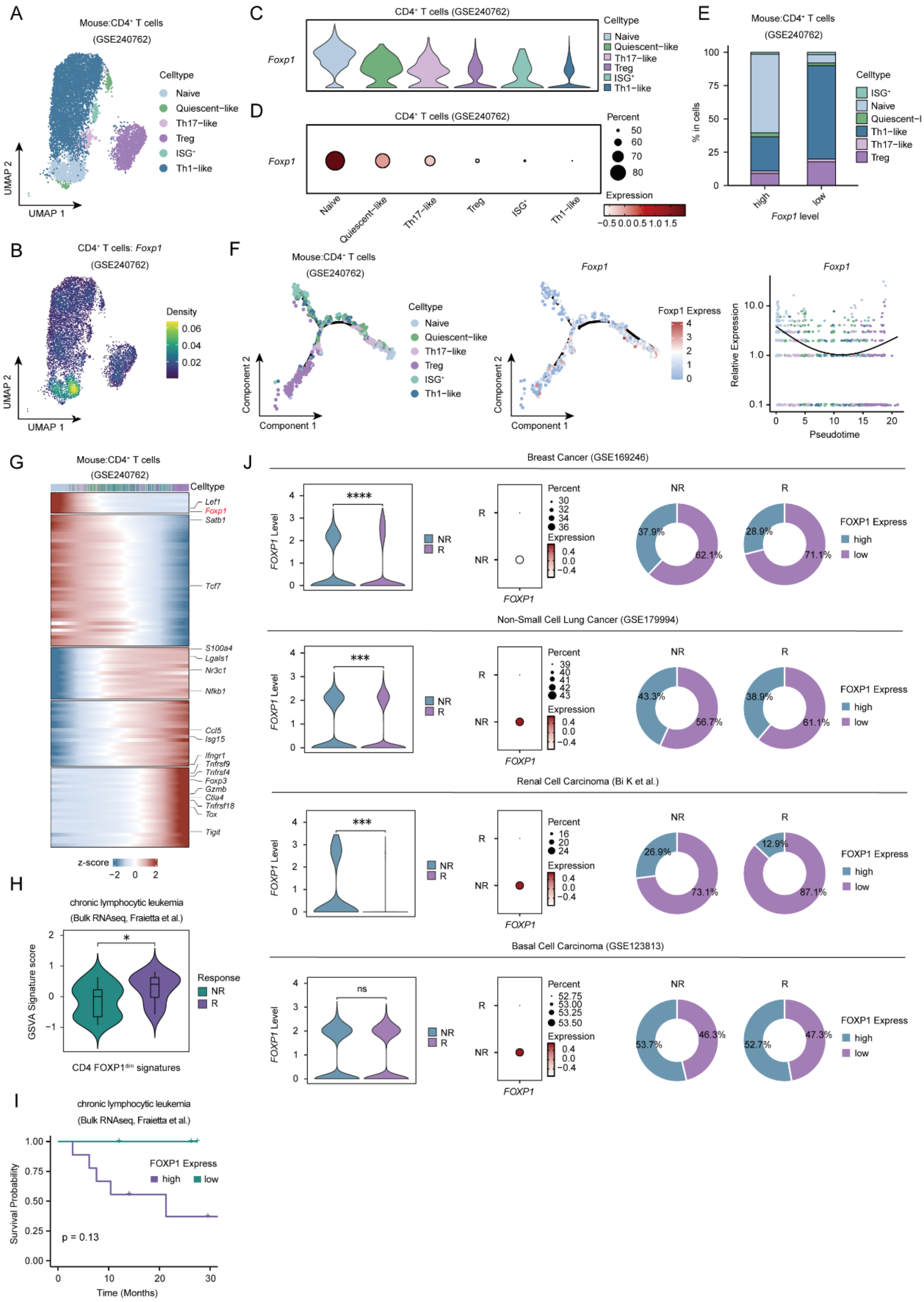

Figure S1. *FOXP1* expression in mouse CD4+ T cells and ICB-treated patients, and its association with survival after CAR-T therapy, related to Figure 1.

(A-G) A total of 21,673 CD4+ T cells derived from murine melanoma were obtained from the GSE240762 scRNA-seq dataset. These are descriptive cell-level visualizations without pseudobulk aggregation or statistical testing. (A) UMAP visualization of CD4+ T-cell subpopulations. (B-D) Density

plot (B), violin plot (C), and dot plot (D) showing *FOXP1* expression across different CD4$^+$ T-cell subpopulations. (E) Cell type composition of FOXP1-high and FOXP1-low groups in CD4$^+$ T cells. Cells were stratified based on normalized FOXP1 expression levels, with a threshold set at 2.0. (F) Pseudotime analysis of CD4$^+$ T cells showing the distribution of different subpopulations along the differentiation trajectory (left), *FOXP1* expression patterns across the trajectory (middle), and dynamic changes of *FOXP1* expression along pseudotime (right). (G) Heatmap showing the top 100 genes with significant expression changes, together with *FOXP1*, in CD4$^+$ T cells across pseudotime. (H-I) Bulk RNA-seq analyses of the chronic lymphocytic leukemia cohort treated with CAR-T cells. (H) FOXP1^{dim} CD4$^+$ T cell signature scores in responders (R) and non-responders (NR). (I) Kaplan–Meier survival analysis of groups defined by *FOXP1* expression level in CAR-T cells. (J) Violin plot and dot plot showing *FOXP1* expression levels in responder (R) and non-responder (NR) patients, and circular plot displaying the proportion of FOXP1-high and FOXP1-low cells, derived from breast cancer (BC), non-small cell lung cancer (NSCLC), renal cell carcinoma (RCC), and basal cell carcinoma (BCC) cohorts treated with immune checkpoint blockade (ICB). These analyses were performed at the cell-level without pseudobulk aggregation. *$p < 0.05$, ** $p < 0.01$, *** $p < 0.001$, **** $p < 0.0001$; two-sided Wilcoxon rank-sum test (H, J); a log-rank test (I).

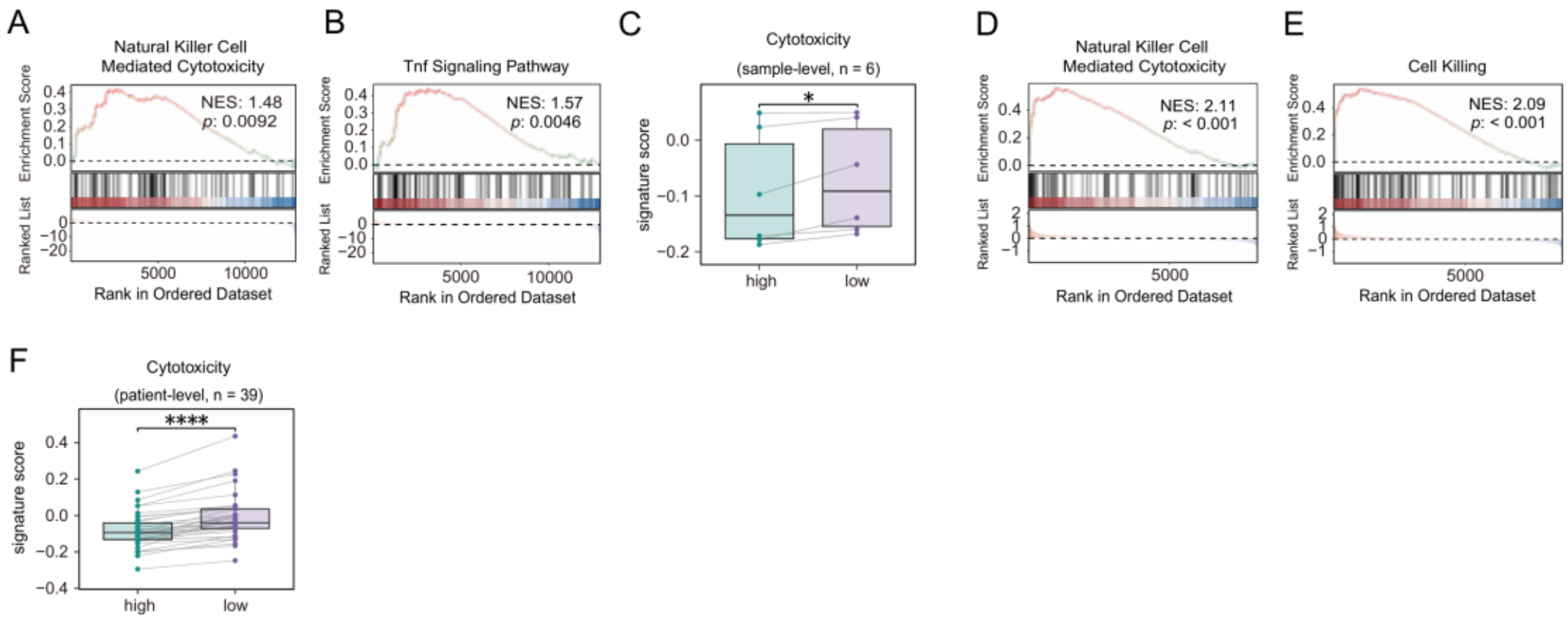

Figure S2. FOXP1-low CD4⁺ T cells exhibit enhanced cytotoxic function, related to Figure 3.

(A-C) Analyses of integrated mouse tumor scRNA-seq datasets (GSE240762, GSE152022, and GSE298152). (A-B) Pseudobulk GSEA of natural killer cell-mediated cytotoxicity and TNF signaling pathway in FOXP1-low versus FOXP1-high $CD4^+$ T cells. (C) Sample-level cytotoxicity signature scores in FOXP1-high and FOXP1-low $CD4^+$ T cells (n = 6). (D-E) Pseudobulk GSEA of natural killer cell-mediated cytotoxicity and cell killing signaling pathway in FOXP1-low versus FOXP1-high $CD4^+$ T cells. (human scRNA-seq data, from Figure 1A) (F) Patient-level cytotoxicity signature scores in FOXP1-high and FOXP1-low $CD4^+$ T cells (n = 39). (human scRNA-seq data, from Figure 1A). *$p < 0.05$, ** $p < 0.01$, *** $p < 0.001$, **** $p < 0.0001$; two-sided Wilcoxon signed-rank test (C, F).

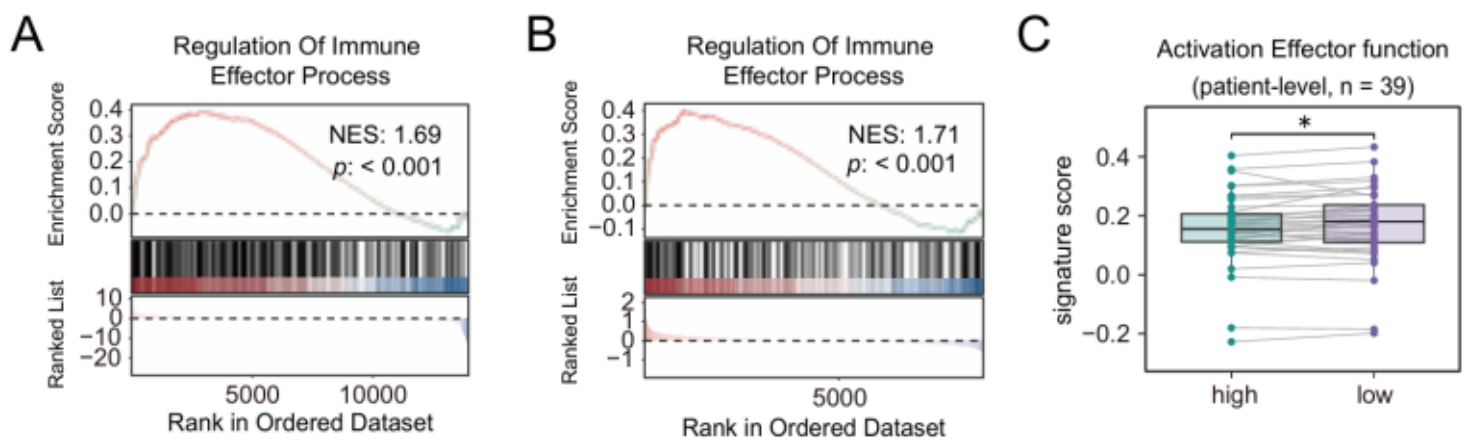

Figure S3. FOXP1-low CD4⁺ T cells exhibit enhanced effector-associated transcriptional programs, related to Figure 4.

(A) Pseudobulk GSEA of the regulation of immune effector process in FOXP1-low versus FOXP1-high $CD4^+$ T cells from integrated mouse tumor scRNA-seq datasets (GSE240762, GSE152022, and GSE298152). (B) Pseudobulk GSEA of the regulation of immune effector process in human $CD4^+$ T cells. (human scRNA-seq data, from Figure 1A) (C) Patient-level activation-effector signature scores in FOXP1-high and FOXP1-low $CD4^+$ T cells. (human scRNA-seq data, from Figure 1A). $*p < 0.05$, $** p < 0.01$, $*** p < 0.001$, $**** p < 0.0001$; two-sided Wilcoxon signed-rank test (C).

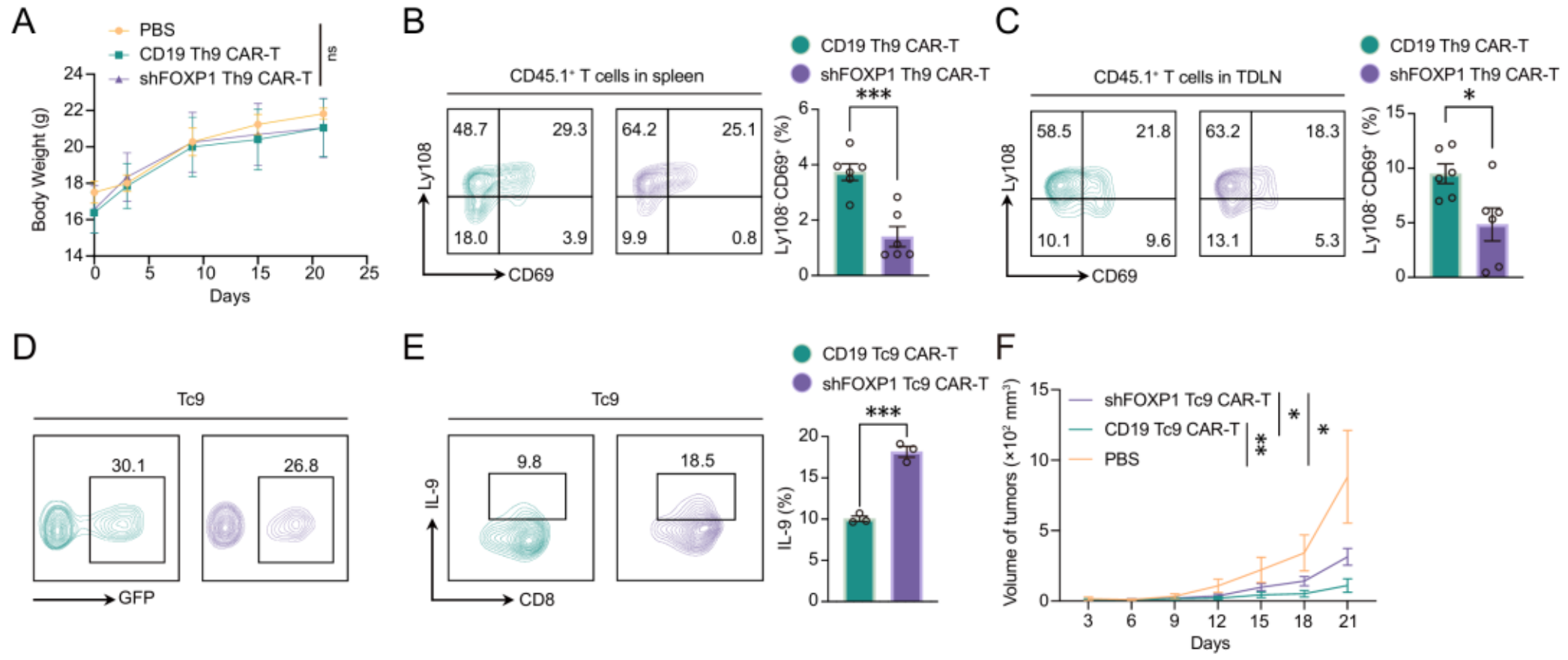

Figure S4. shFOXP1 CAR-T cells exhibited low exhaustion levels *in vivo*, related to Figure 5.

(A) Body weight of mice after Th9 CAR-T treatment. PBS: n=5, CD19 CAR-T/shFOXP1 CAR-T: n=6. Mean ± SEM. (B-C) Representative flow cytometry plots (B: spleen, C: tumor-draining lymph nodes) showing the percentage of $CD69^{+}Ly108^{-}$ (Tex^{term}) among the infused CAR-T cells on day 24 post-treatment. Mean ± SEM, n =6. (D) Flow cytometric analysis of GFP^{+} transduction efficiency following retroviral construction of shFOXP1 Tc9 CAR-T and CD19 Tc9 CAR-T cells. (E) Flow cytometric measurement of interleukin-9 (IL-9) secretion in CD19 Tc9 CAR-T and shFOXP1 Tc9 CAR-T cells, with corresponding quantitative analysis. Mean ± SEM, n = 3. (F) Tumor burden was measured with vernier calipers every 3 days after Tc9 CAR-T cell infusion. Mean ± SEM. PBS: n=5, CD19 CAR-T/shFOXP1 CAR-T: n=6. *$p < 0.05$, ** $p < 0.01$, *** $p < 0.001$, **** $p < 0.0001$.

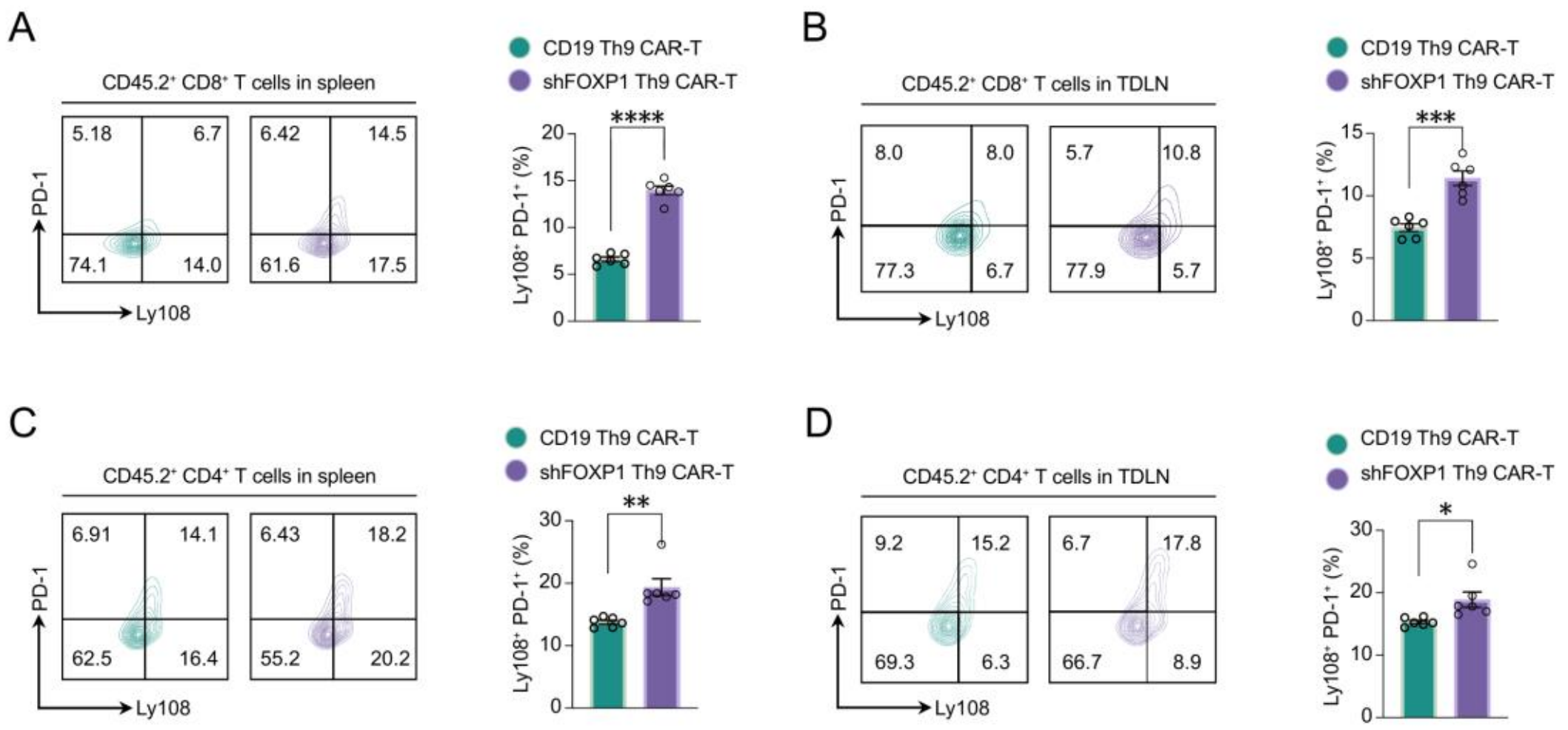

Figure S5. Endogenous T cells in the shFOXP1 CAR-T group acquire a stem-like progenitor exhausted phenotype, related to Figure 6.

(A) Ly108 and PD-1 expression levels in endogenous $CD8^+$ T cells from the spleen. Mean ± SEM, n =6 (B) Ly108 and PD-1 expression levels in endogenous $CD8^+$ T cells from the tumor-draining lymph nodes. Mean ± SEM, n =6 (C) Ly108 and PD-1 expression levels in endogenous $CD4^+$ T cells from the spleen. Mean ± SEM, n =6 (D) Ly108 and PD-1 expression levels in endogenous $CD4^+$ T cells from the tumor-draining lymph nodes. Mean ± SEM, n =6. *$p < 0.05$, ** $p < 0.01$, *** $p < 0.001$, **** $p < 0.0001$.

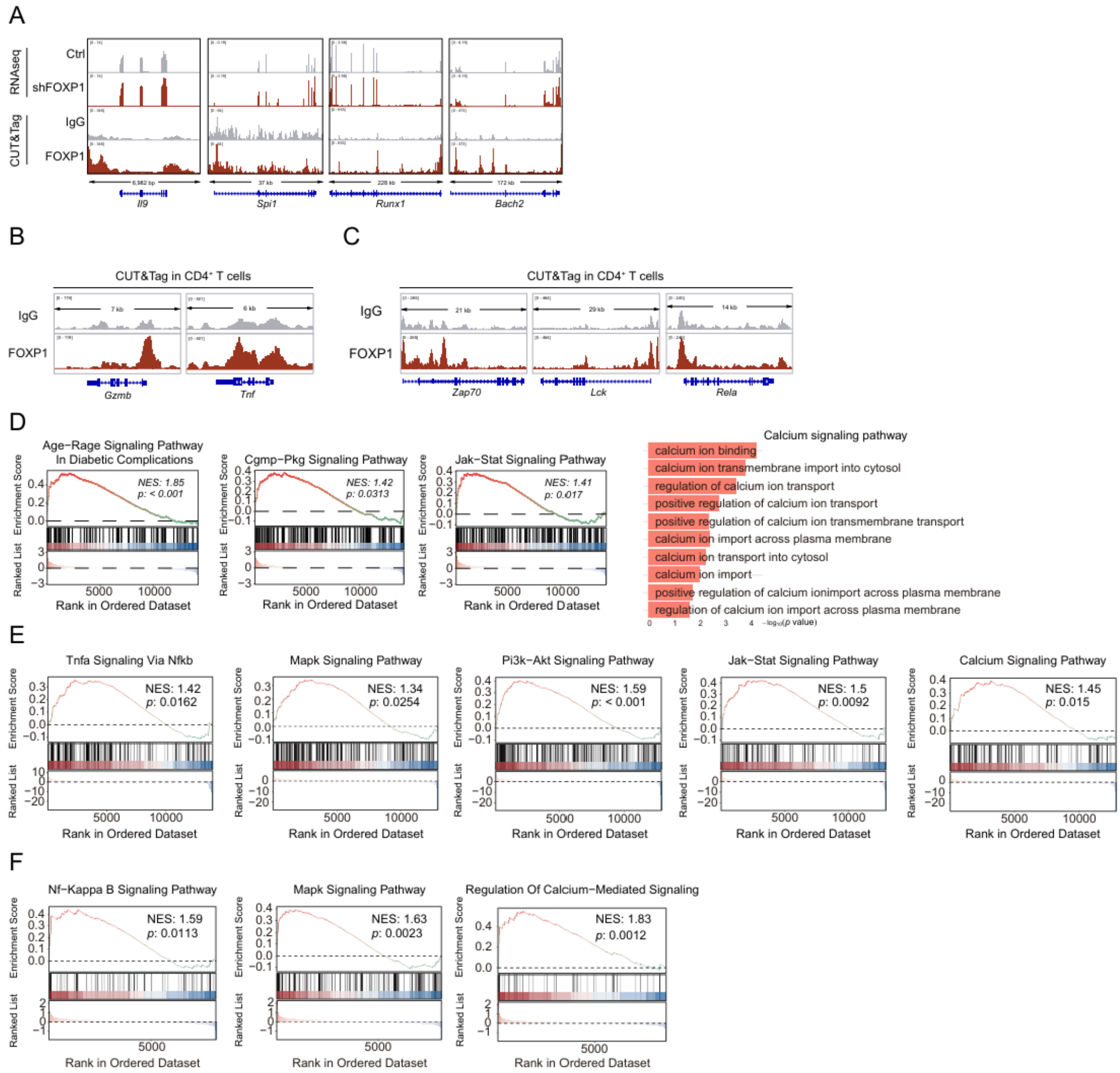

Figure S6. FOXP1 knockdown potentiates the antitumor function of Th9 CAR T cells through coordinated regulation of multiple signaling pathways, related to Figure 8.

(A) RNA-seq and CUT&Tag peak profiles of FOXP1 at the *il9* locus and *il9* expression-related genes (*Spi1*, *Bach2*, and *Runx1*) in CD4$^+$ T cells. (B) CUT&Tag peak profiles of FOXP1 at the *Gzmb* and *Tnf* loci. (C) CUT&Tag peak profiles of FOXP1 at the *Zap70*, *Lck*, and *Rela* loci. (D) GSEA of bulk RNA-seq data comparing shFOXP1 CAR-Th9 with CD19 CAR-Th9 reveals enrichment of the AGE-RAGE, cGMP-PKG, and JAK-STAT signaling pathways, and identifies significantly enriched GO terms associated with the upregulated calcium signaling pathway. (E) Pseudobulk GSEA of the NF-κB, MAPK, PI3K-AKT, JAK-STAT, and calcium signaling pathways in FOXP1-low versus FOXP1-high CD4$^+$ T cells from integrated mouse tumor scRNA-seq datasets (GSE240762, GSE152022, and GSE298152). (F) Pseudobulk GSEA of the NF-κB, MAPK, and calcium signaling pathways in FOXP1-low versus FOXP1-high CD4$^+$ T cells (human scRNA-seq data, from Fig. 1A).
